# Genome-Scale Mapping of *Escherichia coli* σ^54^ Reveals Widespread, Conserved Intragenic Binding

**DOI:** 10.1371/journal.pgen.1005552

**Published:** 2015-10-01

**Authors:** Richard P. Bonocora, Carol Smith, Pascal Lapierre, Joseph T. Wade

**Affiliations:** 1 Wadsworth Center, New York State Department of Health, Albany, New York, United States of America; 2 Department of Biomedical Sciences, School of Public Health, University at Albany, Albany, New York, United States of America; Max Planck Institute for Terrestrial Microbiology, GERMANY

## Abstract

Bacterial RNA polymerases must associate with a σ factor to bind promoter DNA and initiate transcription. There are two families of σ factor: the σ^70^ family and the σ^54^ family. Members of the σ^54^ family are distinct in their ability to bind promoter DNA sequences, in the context of RNA polymerase holoenzyme, in a transcriptionally inactive state. Here, we map the genome-wide association of *Escherichia coli* σ^54^, the archetypal member of the σ^54^ family. Thus, we vastly expand the list of known σ^54^ binding sites to 135. Moreover, we estimate that there are more than 250 σ^54^ sites in total. Strikingly, the majority of σ^54^ binding sites are located inside genes. The location and orientation of intragenic σ^54^ binding sites is non-random, and many intragenic σ^54^ binding sites are conserved. We conclude that many intragenic σ^54^ binding sites are likely to be functional. Consistent with this assertion, we identify three conserved, intragenic σ^54^ promoters that drive transcription of mRNAs with unusually long 5ʹ UTRs.

## Author Summary

Bacterial RNA polymerases must associate with a σ factor to bind to promoter DNA sequences upstream of genes and initiate transcription. There are two families of σ factor: σ^70^ and σ^54^. Members of the σ^54^ family are distinct from members of the σ^70^ family in their ability to bind promoter DNA sequences, in association with RNA polymerase, in a transcriptionally inactive state. We have determined positions in the *Escherichia coli* genome that are bound by σ^54^, the archetypal member of the σ^54^ family. Surprisingly, we identified 135 binding sites for σ^54^, a huge increase over the number of previously described sites. Our data suggest that there are more than 250 σ^54^ sites in total. Strikingly, most σ^54^ binding sites are located inside genes, whereas only one intragenic σ^54^ binding site has previously been described. The location and orientation of intragenic σ^54^ binding sites is non-random, and many intragenic σ^54^ binding sites are conserved in other bacterial species. We conclude that many intragenic σ^54^ binding sites are likely to be functional. Consistent with this notion, we identify three σ^54^ promoters in *E*. *coli* that are located inside genes but drive transcription of unusual mRNAs for the neighboring genes.

## Introduction

Transcription initiation, the first step in gene expression, is highly regulated to ensure correct timing of developmental processes and the response to environmental stimuli. In bacteria, transcription initiation involves association of RNA polymerase (RNAP) with promoter DNA. Core RNAP must associate with a Sigma (σ) factor to make sequence-specific contacts with promoter DNA [1]. Following promoter escape, σ factors are released from the elongating RNAP [2].

Bacterial cells often express a single “primary” σ factor and multiple “alternative” σ factors. The primary σ factor is constitutively active and is responsible for transcription of most genes. Alternative σ factors are typically expressed or activated under specific growth conditions and recognize promoters with nucleotide sequences distinct from those recognized by the primary σ factor [3]. Consequently, alternative σ factors govern the transcription of different sets of genes (regulons). Depending on the growth phase, environmental conditions, and developmental stage experienced by the cell, the composition of the pool of active σ factors can vary, allowing for dynamic and rapid expression of different regulons as needed. *Escherichia coli* has one primary σ factor (σ^70^) and six alternative σ factors (σ^19^, σ^24^, σ^28^, σ^32^, σ^38^ and σ^54^) [3].

There are two families of σ factor in bacteria: the σ^70^ family and the σ^54^ family. σ^54^ proteins differ dramatically from those in the σ^70^ family, both in sequence and domain structure. σ^54^ promoter elements consist of conserved nucleotides located at -12 and -24 with respect to the transcription start site [4]. This contrasts with members of the σ^70^ family, which recognize conserved promoter elements located at roughly -10 and -35 with respect to the transcription start site [3]. Unlike the members of the σ^70^ family, σ^54^ proteins have been shown to bind promoter DNA independent of core RNAP *in vitro* [5]. Another distinguishing characteristic of σ^54^ proteins is their absolute requirement for activator proteins, known as bacterial enhancer binding proteins (bEBPs), to initiate transcription [4,6]. bEBPs act in a manner distinct from typical σ^70^ transcriptional activator proteins: rather than helping to recruit RNAP, like most activators of σ^70^, bEBPs use ATP hydrolysis to drive isomerization of RNAP already bound at the promoter [4]. Thus, both active and inactive forms of RNAP:σ^54^ are bound at promoters. The archetypal member of the σ^54^ family is σ^54^ from *E*. *coli*. Originally identified as a regulator of genes involved in nitrogen metabolism and assimilation under nitrogen limiting conditions [7], *E*. *coli* σ^54^ has since been shown to play important regulatory roles in a variety of other cellular processes. Similarly, σ^54^ homologues in other species regulate a wide range of processes, including flagellar synthesis and virulence [8]. *E*. *coli*, and most other bacterial species, have only one σ^54^ family protein [9].

Genome-wide DNA binding studies have been performed for a handful of σ factors in different bacteria. σ factors show a wide range in the number of sites bound, from just a few sites for extracytoplasmic function (ECF) σ^70^ family factors [10,11] to more than a thousand sites for *E*. *coli* σ^70^ [12,13]. These studies have revealed new promoters and often help to clarify gene expression changes that are a direct result of proximal RNAP:σ binding. Unexpectedly, some σ factors have been shown to bind extensively inside genes [14,15,16,17,18]. Notably, 58% of the σ^54^ binding sites described in *Salmonella* Typhimurium occur inside genes [14]. Interestingly, in *Vibrio cholerae*, only 10% of the described σ^54^ binding sites occur inside genes [19], suggesting variation in the regulatory capacity of σ^54^ across bacterial species. The function, if any, of σ^54^ binding events inside genes is unknown, with the exception of one promoter that has been shown to drive transcription of an mRNA for the downstream gene, with the RNA having an unusually long 5ʹ UTR [20].

In this study we use chromatin immunoprecipitation (ChIP) followed by deep sequencing (ChIP-seq) to determine the genome-wide binding profile of σ^54^ in *E*. *coli*. We confirm all but one previously reported promoter and identify 116 novel, high confidence σ^54^ binding sites. Notably, two thirds of the σ^54^ binding sites occur inside genes. These intragenic sites are oriented non-randomly, with the majority oriented in the same direction as the overlapping gene. We show that three intragenic binding sites are functional promoters, and conservation analysis suggests that many others are functional under different growth conditions.

## Results

### Genome-scale identification of σ^54^ binding sites using ChIP-seq

**Fig 1:**
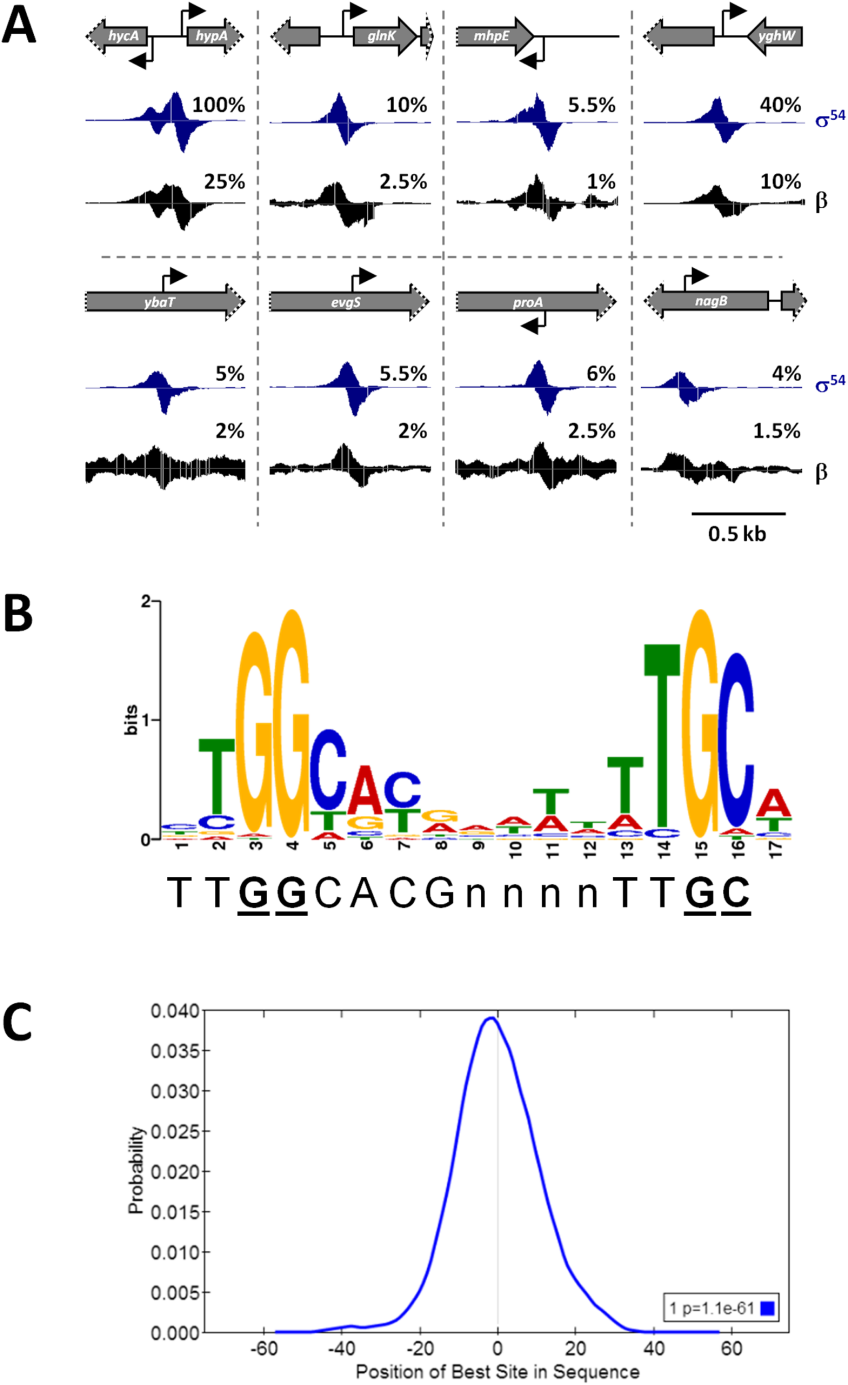
ChIP-seq identifies σ^54^ binding sites on a genomic scale. **(A)** Examples of σ^54^ and RNAP (β) binding. Schematics depict the local genomic environment surrounding selected σ^54^ binding sites identified by ChIP-seq. Grey arrows represent genes. Grey arrows with dotted lines indicate that only a portion of the gene is shown. Bent, black arrows indicate the location and direction of σ^54^ binding motifs associated with identified ChIP-seq peaks. Histograms show mapped sequence reads from σ^54^ (blue) and β (black) ChIP-seq experiments. Percentages indicate relative scale on the y-axis. **(B)** Consensus motif derived from 135 σ^54^ ChIP-seq peaks, determined with MEME (E-value = 1.8e^-213^). The established σ^54^ consensus sequence [6] is shown beneath the logo. Nucleotides in bold, underlined text are those most important for σ^54^ binding [6]. **(C)** Centrimo analysis of σ^54^ motifs identified by MEME, showing the position of the motifs relative to the ChIP-seq peak centers. The graph indicates the average density of motif position for all 135 motif-containing regions, using 10 bp bins from position -75 to +75 relative to the σ^54^ ChIP-seq peak.

**Fig 2:**
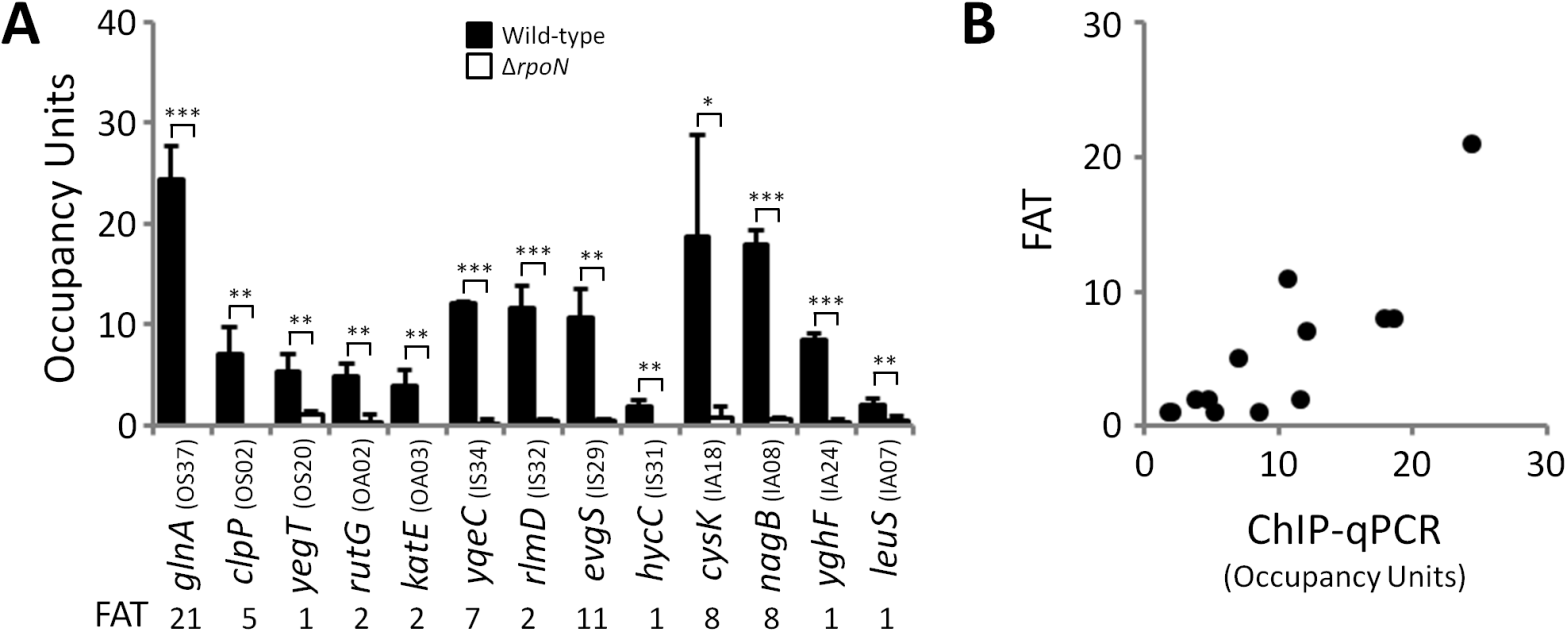
ChIP-seq enrichment represents genuine σ^54^ binding. **(A)** Targeted validation of σ^54^ binding sites. ChIP-qPCR measurement of σ^54^ binding at putative sites identified by ChIP-seq in wild-type (MG1655; black bars) and Δ*rpoN* (RPB146; white bars) *E*. *coli* strains. The cognate promoter IDs and the fold above threshold (FAT; see Methods) scores (Tables 1 & 2) are indicated in parentheses and below the gene name, respectively. Gene names for “outside sense” (OS) and “outside antisense” (OA) binding sites correspond to the first gene downstream of the binding site (downstream relative to the orientation of the binding site). Gene names for “inside sense” (IS) and “inside antisense” (IA) binding sites correspond to the gene that contains the binding site. Occupancy units represent background-subtracted enrichment of target regions relative to a control region within the transcriptionally silent gene *bglB*. Error bars represent the standard deviation from three independent biological replicates. Significant differences between wild-type and Δ*rpoN* values are indicated (*p ≤ 0.05, **p ≤ 0.01, ***p ≤ 0.001). **(B)** Correlation of ChIP-qPCR and ChIP-seq data. Values obtained from ChIP-qPCR (occupancy units) and ChIP-seq (FAT) using the σ^54^ antibody in *E*. *coli* were compared.

We predicted that all σ^54^-transcribed promoters would be bound by RNAP:σ^54^ under all growth conditions in which σ^54^ is expressed. Therefore, we mapped the binding of σ^54^ using ChIP-seq for cells grown to mid-logarithmic phase in M9 minimal medium. Using a high stringency analysis, we identified 145 ChIP-seq peaks that correspond to putative sites of σ^54^ binding (Tables 1 and 2 and S1 Table). The identified peaks have a bimodal shape, typical of ChIP-seq data (Fig 1A) [21]. We used MEME [22] to identify enriched sequence motifs in the 150 bp regions surrounding each of the putative σ^54^ binding sites. A motif closely resembling the known σ^54^–24/-12 promoter elements [6] was identified in 135 of the regions (Fig 1B). This represents a highly significant enrichment for the motif within these sequences (MEME E-value 1.8e^-213^). Furthermore, the distribution of motif positions within the ChIP-based query sequences was non-random: motifs were far more likely to be located at the center of the query sequence than expected by chance (*p* = 1.1e^-61^; Fig 1C), as would be expected for genuine σ^54^ binding sites. ChIP-seq peaks without an associated motif are listed in S1 Table. We selected 13 putative σ^54^ binding sites for validation. All of these sites are associated with a motif identified by MEME. We used quantitative PCR to measure enrichment of these sites by ChIP (ChIP-qPCR) of σ^54^ in wild type cells or cells in which *rpoN* is deleted. In all cases, we detected robust enrichment in wild type cells that was significantly higher than that in Δ*rpoN* cells (Fig 2A). Moreover, ChIP-qPCR enrichment scores correlated well with enrichment measured by ChIP-seq (R^2^ = 0.71; Fig 2B). We also selected five of the ten regions for which we detected a ChIP-seq peak but no motif. We suspected that these were false positives that are commonly found in ChIP-seq datasets for regions that are highly transcribed [12,23,24,25]. We used ChIP-qPCR of σ^54^ in wild type cells and Δ*rpoN* cells. We observed no significant difference in enrichment levels between the wild type and Δ*rpoN* cells (S1 Fig), consistent with these sites being false positives. We conclude that nearly all of the binding sites identified by ChIP-seq, for which we also detected a motif, represent genuine sites of σ^54^ binding. For all further analyses, we required that the ChIP-enriched sequences include a σ^54^ promoter motif identified by MEME to be called as a genuine σ^54^ binding site (Tables 1 and 2). We refer to these 135 binding sites as “high stringency sites”. Note that we use the term “binding site” rather than “promoter” because we do not know which sites represent functional promoters, as opposed to σ^54^ binding sites that never lead to productive transcription.

**Table 1:** Intergenic σ^54^ ChIP-seq peaks.

						Downstream				
**ID** ^a^ ^,^ ^b^	**Peak Center** ^c^	**FAT** ^d^	**Motif** ^e^	**Motif Center** ^f^	**Motif Strand** ^g^	**b#** ^h^	**Gene** ^h^	**Distance** ^i^	**PSSM** ^j^	**Ecocyc** ^k^
**Intergenic Sense**										
OS01	347850	17	G**TGGC**ACACCCC**TTGC**T	347851	+	b0331	*prpB*	36	9.469	P
OS02	455741	5	T**TG**T**C**ATGAATT**TTGC**A	455728	+	b0437	*clpP*	154	10.247	
OS03	471768	18	C**TGGC**ACACCGC**TTGC**A	471761	+	b0450	*glnK*	42	10.502	P
OS04	688441	21	T**TGGC**ACATCTA**TTGC**T	688459	-	b0656	*insH3*	204	11.713	P
OS05	815921	4	T**TGGC**AGGTTAA**TTGC**T	815934	-	b0780	*ybhK*	45	10.111	P
OS06	847290	6	C**TGGC**ACGATTT**TT**T**C**A	847290	-	b0811	*glnH*	44	10.791	C
OS07	882891	10	T**TGGC**GAAGAAA**TTGC**A	882888	+	b0846	*rcdA*	3739	10.538	
OS08	892944	2	TAT**GC**ACGTTTA**TTGC**A	892939	+	b0854	*potF*	49	6.829	P
OS09	1014824	4	G**TGGC**GTGAATT**TTGC**G	1014827	+	b0953	*rmf*	92	10.773	
OS10	1073263	6	C**TGGC**ATCCGCT**TTGC**A	1073271	-	b1012	*rutA*	18	9.963	P
OS11	1165190	4	T**TGG**TATGACCAA**TGC**A	1165182	+	b1109	*ndh*	107	9.468	
OS12	1271626	2	G**TGG**TCCGTGGA**TTGC**A	1271626	+	b1218	*chaC*	85	6.093	P
OS13	1308544	22	AG**GGC**ACGGTTT**TTGC**A	1308566	-	b1250	*kch*	254	11.713	P
OS14	1328870	3	C**TGGC**AATAGAT**TTGC**T	1328862	+	b1274	*topA*	191	9.551	
OS15	1366050	21	T**TGGC**ACGCAAA**TTG**TA	1366043	+	b1304	*pspA*	41	10.355	C
OS16	1561149	10	A**TGGC**ATGAGATC**TGC**A	1561153	-	b1488	*ddpX*	34	10.511	P
OS17	1830088	7	C**TGGC**ACGAACCC**TGC**A	1830087	-	b1748	*astC*	62	11.294	C
OS18	1864820	6	A**TGGC**ATGAGAG**TTGC**T	1864820	+	b1783	*yeaG*	93	10.573	P
OS19	2060018	24	C**TGGC**AAGCATC**TTGC**A	2060021	-	b1988	*nac*	45	11.669	C
OS20	2176637	1	C**TGGC**CCGCCTT**TTGC**G	2176641	+	b2098	*yegT*	183	9.132	
OS21	2321425	5	C**TGGC**ACTCCCC**TTGC**T	2321415	+	b2221	*atoD*	35	9.39	C
OS22	2425886	12	A**TGGC**ATAAGACC**TGC**A	2425890	-	b2310	*argT*	58	9.457	P
OS23	2599175	16	A**TGGC**ATCCTTTA**TGC**A	2599173	+	b2481	*hyfA*	31	10.524	C
OS24	2689367	200	T**TGGC**ACAGTTAC**TGC**A	2689382	-	b4441	*glmY*	1	12.388	C
OS25	2830446	11	C**TGGC**ACGCAATC**TGC**A	2830442	+	b2710	*norV*	37	12.266	C
OS26	2836164	11	C**TGGC**ATGATTTG**TG**AA	2836173	-	b2713	*hydN*	27	9.175	C
OS27	2848494	91	T**TGGC**ACAAAAAA**TGC**T	2848502	-	b2725	*hycA*	26	12.361	C
OS28	2848633	201	C**TGGC**ACAATTA**TTGC**T	2848630	+	b2726	*hypA*	20	14.094	C
OS29	2998270	11	C**TGGC**GTAAATC**TTGC**C	2998264	+	b2866	*xdhA*	84	10.928	C
OS30	3004190	3	C**TGGC**ACACTTA**TTG**TT	3004185	+	b2870	*ygeW*	80	9.837	
OS31	3014019	26	G**TGG**TGCGATTG**TTGC**T	3014001	+	b2878	*ygfK*	62	10.064	P
OS32	3029005	3	TAT**GC**CCGTTTA**TTGC**A	3029008	-	b2887	*ygfT*	36	4.529	
OS33	3217457	10	G**TGGC**GCAATCCC**TGC**A	3217461	+	b3073	*patA*	36	8.711	C
OS34	3416975	29	C**TGGC**ACTACTT**TTGC**T	3416975	+	b3268	*yhdW*	70	12.521	P
OS35	3556144	17	C**TGGC**ACGACGG**TTGC**A	3556148	-	b3421	*rtcB*	28	11.161	C
OS36	3598850	47	C**TGGC**ACAGTTG**TTGC**T	3598855	-	b3461	*rpoH*	30	12.987	C
OS37	4056144	21	T**TGGC**ACAGATT**T**C**GC**T	4056149	-	b3870	*glnA*	73	11.277	C
OS38	4199750	39	T**TGGC**ACGGAAGA**TGC**A	4199755	-	b4002	*zraP*	25	12.296	P
OS39	4199910	9	A**TGGC**ATGATTTC**TGC**T	4199909	+	b4003	*zraS*	21	11.395	P
OS40	4260832	15	T**TGGC**ATGATTC**TTG**TA	4260819	+	b4050	*pspG*	25	10.745	C
OS41	4297443	41	G**TGGC**ATAAAAGA**TGC**A	4297449	-	b4079	*fdhF*	41	10.976	C
OS42	4437375	34	C**TGGC**ATCACAC**TTGC**G	4437375	-	b4216	*ytfJ*	71	9.299	
**ID** ^a^ ^,^ ^b^	**Peak Center** ^c^	**FAT** ^d^	**Motif** ^e^	**Motif Center** ^f^	**Motif Strand** ^g^	**b#** ^h^	**Gene** ^h^	**Distance** ^i^	**PSSM** ^j^	
**Intergenic Antisense**										
OA01	374140	12	TG**GGC**ATACAAAA**TGC**A	374132	-	b0346	*mhpR*	6496	9.097	
OA02	1067669	2	CC**GGC**ATGAACAA**TGC**G	1067678	+	b1013	*rutR*	5796	8.231	
OA03	1814362	2	GC**GGC**GTGAACC**TTGC**A	1814382	-	b1731	*cedA*	2675	9.191	
OA04	2802707	12	G**TGGC**ATGAATA**TTG**AT	2802718	-	b2671	*ygaC*	4691	10.229	
OA05	3144330	61	C**TGGC**ATATATT**TTGC**C	3144316	+	b3001	*gpr*	1589	11.588	
OA06	3370638	3	T**TGG**TATGAAAA**TTG**TA	3370640	+	b3227	*dcuD*	2253	9.468	
OA07	3809831	1	G**TGGC**GTAGTATAC**GC**T	3809844	+	b3639	*dfp*	923	7.219	
OA08	3889556	3	A**TGGC**TGGCTTC**TTG**AA	3889519	-	b3702	*dnaA*	7804	4.934	

^a^ Unique ID; OS = Outside of a gene in the Sense orientation, OA = Outside of a gene in the Antisense orientation; each peak is assigned a unique number for cross-referencing with other datasets

^b^ Underlined text indicates that a σ^54^ has been identified in the homologous position in *Salmonella* Typhimurium [14]

^c^ Genome coordinate (U00096.2) of ChIP-seq peak center

^d^ FAT = Fold Above Threshold score (indication of ChIP-seq occupancy)

^e^ Associated motif identified using MEME; consensus positions indicated in bold

^f^ Genome coordinate (U00096.2) of motif center

^g^ Genomic orientation of associated motif

^h^ Closest, appropriately oriented, downstream gene (from the predicted transcription start site, 19 bp downstream of the motif center)

^i^ Distance from the predicted transcription start site (19 bp downstream of the motif center) to the start of the closest, appropriately oriented, downstream gene (bp); note that for OS07, the ChIP-seq peak is intergenic but the predicted transcription start site is intragenic

^j^ Position Specific Scoring Matrix (PSSM) score (indication of similarity to the consensus site; see Methods)

^k^ P = Promoter is Predicted in Ecocyc; C = promoter is experimentally Confirmed in Ecocyc [26]

**Table 2:** Intragenic σ^54^ ChIP-seq peaks.

						Overlapping			Downstream			
**ID** ^a^ ^**,**^ ^b^	**Peak Center** ^c^	**FAT** ^d^	**Motif** ^e^	**Motif Center** ^f^	**Motif Strand** ^g^	**b#** ^h^	**Gene** ^h^	**Strand** ^i^	**b#** ^j^	**Gene** ^j^	**Distance** ^k^	**PSSM** ^l^
**Intragenic Sense**												
IS01	33273	2	C**TGGC**CTTCGAA**TTGC**A	33270	+	b0033	*carB*	+	b0034	*caiF*	1027	8.626
IS02	512538	7	C**TGGC**ACTGGTT**TTGC**T	512537	+	b0486	*ybaT*	+	b0487	*cueR*	679	12.126
IS03	534346	2	GC**GGC**ACAAATGC**TGC**A	534345	+	b0507	*gcl*	+	b0508	*hyi*	588	9.457
IS04	551087	1	C**TGGC**ACCGCGTG**TGC**A	551095	-	b0522	*purK*	-	b0517	*allD*	5500	7.934
IS05	567582	2	G**TGG**TGCAATAC**TTGC**A	567568	+	b0543	*emrE*	+	b0544	*ybcK*	543	10.546
IS06	655861	1	T**TGG**TAAAGTTT**TTGC**T	655846	+	b0622	*pagP*	+	b0623	*cspE*	654	10.387
IS07	769197	2	CC**GG**TATGGAATA**TGC**T	769192	+	b0732	*mngB*	+	b0733	*cydA*	1484	9.777
IS08	773450	1	TG**GG**AACGCTTC**TTGC**C	773449	+	b4515	*cydX*	+	b0735	*ybgE*	82	6.849
IS09	808913	1	C**TGG**AACAAATGG**TGC**A	808911	+	b0775	*bioB*	+	b0776	*bioF*	691	8.797
IS10	939112	1	C**TGGC**CTCGACT**TTGC**A	939098	+	b0893	*serS*	+	b0894	*dmsA*	1070	9.624
IS11	1037161	2	TC**GG**TATCAATT**TTGC**T	1037146	+	b0978	*appC*	+	b0979	*appB*	1358	9.873
IS12	1177855	1	G**TGG**AACAAAAA**TTGC**G	1177846	+	b1119	*nagK*	+	b1120	*cobB*	999	9.052
IS13	1213765	6	T**TGGC**GCAGGTT**TTGC**T	1213771	-	b1163	*bluF*	-	b1162	*bluR*	483	11.673
IS14	1247330	3	TCA**GC**ATGAACA**TTGC**A	1247325	-	b1198	*dhaM*	-	b1197	*treA*	731	7.778
IS15	1252922	1	T**TGGC**TCAACACA**TGC**A	1252946	-	b1202	*ycgV*	-	b1200	*dhaK*	2861	9.112
IS16	1462952	5	T**TGGC**ATGGAAAAA**GC**A	1462947	+	b1400	*paaY*	+	b4492	*ydbA*	464	8.192
IS17	1464737	2	CC**GG**TACGGAAA**TTGC**T	1464730	+	b1401	*ydbA_1*	+	b1404	*insI-2*	2528	11.092
IS18	1519002	23	TC**GGC**ATGAATA**TTGC**G	1519010	-	b1451	*yncD*	-	b1448	*mnaT*	2132	10.795
IS19	1535850	2	C**TGGC**ACTACCG**TTGC**A	1535858	-	b1467	*narY*	-	b1466	*narW*	517	10.363
IS20	1615388	6	T**TGG**TGTGGCTT**TTGC**A	1615385	+	b1528	*ydeA*	+	b1530	*marR*	1756	11.186
IS21	1662480	2	TA**GG**AATGGCTA**TTGC**A	1662473	+	b1590	*ynfH*	+	b1591	*dmsD*	50	8.355
IS22	1679126	1	A**TGG**ACTGATTAA**TGC**A	1679141	+	b1606	*folM*	+	b1608	*rstA*	1057	7.976
IS23	1838205	3	G**TGGC**GCAGATTA**TGC**T	1838213	+	b1757	*ynjE*	+	b1759	*nudG*	1309	11.026
IS24	1958599	3	TC**GG**TATGCTGA**TTGC**A	1958590	+	b1876	*argS*	+	b1877	*yecT*	1397	8.616
IS25	2079713	7	AC**GG**TGCAAATT**TTGC**A	2079714	-	b2010	*dacD*	-	b2009	*sbmC*	427	10.137
IS26	2101814	2	G**TGG**TACAGAAAA**TGC**G	2101819	-	b2032	*wbbK*	-	b4571	*wbbL*	401	8.622
IS27	2360527	2	A**TGGC**ACTGAATA**TGC**T	2360543	-	b2249	*yfaY*	-	b2248	*rhmR*	294	10.808
IS28	2370326	2	C**TGGC**ATGGAGCC**TGC**A	2370324	+	b2257	*arnT*	+	b4544	*arnE*	253	10.155
IS29	2484404	11	C**TGGC**ATACATTA**TGC**A	2484401	+	b2370	*evgS*	+	b2376	*ypdI*	8316	12.682
IS30	2526524	6	T**TGGC**ATTGTCG**TTGC**A	2526536	-	b2411	*ligA*	-	b4546	*ypeB*	343	10.476
IS31	2846034	1	G**TGGC**GCGTTTG**TTGC**C	2846045	-	b2723	*hycC*	-	b2722	*hycD*	600	8.895
IS32	2912348	2	C**TGG**AACGCTTT**T**C**GC**A	2912360	-	b2785	*rlmD*	-	b2784	*relA*	675	9.449
IS33	2954738	2	C**TGGC**ACGCGATG**TGC**A	2954753	-	b2821	*ptrA*	-	b2820	*recB*	713	10.167
IS34	3012660	7	TGA**GC**ACGAACC**TTGC**A	3012670	-	b2876	*yqeC*	-	b2875	*yqeB*	399	6.768
IS35	3074948	13	C**TGGC**GGCAATA**TTGC**A	3074950	-	b4465	*yggP*	-	b2930	*yggF*	744	10.066
IS36	3169588	4	T**TGG**TGCGAAAT**TTGC**T	3169579	+	b3026	*qseC*	+	b3028	*mdaB*	964	11.709
IS37	3178185	1	GC**GGC**GCGGGAT**TTGC**A	3178183	+	b3037	*ygiB*	+	b3038	*ygiC*	258	10.055
IS38	3206700	2	A**TGGC**ACCAAAC**TTGC**T	3206687	+	b3063	*ttdT*	+	b3065	*rpsU*	2103	11.358
IS39	3241894	1	T**TGG**TGCCGAATA**TGC**A	3241908	-	b3092	*uxaC*	-	b3091	*uxaA*	558	9.54
IS40	3330429	6	T**TGGC**ATGATGG**TTGC**C	3330421	-	b3184	*yhbE*	-	b3183	*obgE*	653	9.51
IS41	3449718	1	C**TGGC**ATGATTCG**TG**AA	3449703	-	b3319	*rplD*	-	b3317	*rplB*	12	8.485
IS42	3538782	1	G**TGGC**ACTGAACA**TGC**T	3538785	+	b3409	*feoB*	+	b3410	*feoC*	1968	10.166
IS43	3565544	49	AA**GGC**ATGTTTTA**TGC**A	3565552	-	b3429	*glgA*	-	b3428	*glgP*	940	8.839
IS44	3683845	1	T**TGGC**ACGGCAA**TTG**AT	3683854	-	b3530	*bcsC*	-	b3529	*yhjK*	204	9.756
IS45	3803524	7	A**TGG**TGCGTAAAA**TGC**A	3803521	-	b3630	*waaP*	-	b3629	*waaS*	385	8.867
IS46	3966932	10	C**TGG**TGCTCTTT**TTGC**T	3966916	+	b3785	*wecA*	+	b3785	*wzzE*	122	10.016
IS47	4079719	3	C**TGGC**GCGAATTC**TGC**A	4079722	-	b3892	*fdoI*	-	b3891	*fdhE*	468	12.331
IS48	4131399	3	T**TGGC**GCGAATA**TTGC**C	4131401	+	b3941	*metF*	+	b3942	*katG*	459	11.876
IS49	4342113	8	TG**GG**TATGGCTC**TTGC**T	4342109	+	b4120	*melB*	+	b4126	*yjdI*	7753	8.401
IS50	4370368	2	TG**GG**TATCAAAG**TTGC**A	4370368	+	b4143	*groL*	+	b4144	*yjeI*	464	7.795
IS51	4445887	1	CA**GGC**ACTGGAT**TTGC**T	4445887	+	b4221	*tamB*	+	b4222	*ytfP*	30	8.931
IS52	4457537	2	AA**GG**AACTATTC**TTGC**A	4457513	+	b4236	*cybC*	+	b4702	*mgtL*	7917	7.424
IS53	4547262	1	C**TGGC**TCATTAA**TTGC**C	4547266	+	b4320	*fimH*	+	b4322	*uxuA*	2397	8.64
IS54	4561465	3	AC**GGC**AAAGAAA**TTGC**A	4561473	-	b4333	*yjiK*	-	b4332	*yjiJ*	767	9.461
IS55	4606188	5	A**TGGC**AACAAAT**TTGC**A	4606196	+	b4373	*holD*	+	b4374	*yjjG*	20	10.399
IS56	4612117	3	C**TGGC**ATCGTTA**TTGC**T	4612106	+	b4378	*yjjV*	+	b4381	*deoC*	3229	12.389
IS57	4613489	17	C**TGGC**TCTGTTT**TTGC**A	4613489	-	b4379	*yjjW*	-	b4371	*rsmC*	7766	11.411
IS58	4627943	2	C**TGG**AACGCTTCC**TGC**A	4627937	-	b4391	*ettA*	-	b4387	*ytjB*	5131	9.616
**ID** ^a^ ^**,**^ ^b^	**Peak Center** ^c^	**FAT** ^d^	**Motif** ^e^	**Motif Center** ^f^	**Motif Strand** ^g^	**b#** ^h^	**Gene** ^h^	**Strand** ^i^	**b#** ^j^	**Gene** ^j^	**Distance** ^k^	**PSSM** ^l^
**Intragenic Antisense**												
IA01	71210	1	CC**GGC**ACGAAAC**T**C**GC**T	71214	-	b0064	*araC*	+	b0063	*araB*	1147	9.354
IA02	220685	1	T**TGGC**GTCGATA**T**C**GC**C	220686	+	b0197	*metQ*	-	b0200	*gmhB*	2128	6.941
IA03	261344	12	AC**GGC**ACAGTTTA**TGC**A	261348	-	b0243	*proA*	+	b0241	*phoE*	2005	11.216
IA04	453263	1	TC**GGC**ACCATTAA**TGC**T	453248	+	b0434	*yajG*	-	b0435	*bolA*	429	10.001
IA05	485009	2	T**TGGC**GCGTTTC**TTGC**G	485018	-	b0464	*acrR*	+	b0463	*acrA*	156	9.853
IA06	619311	1	T**TGGC**CCGATAA**TTGC**C	619307	+	b0588	*fepC*	-	b0591	*entS*	2197	10.286
IA07	674001	1	GC**GG**TATTGCTC**TTGC**A	673997	+	b0642	*leuS*	-	b0643	*ybeL*	225	8.124
IA08	702665	8	C**TGGC**CTGCTTTA**TGC**A	702663	+	b0678	*nagB*	-	b0679	*nagE*	485	10.647
IA09	797417	4	CCA**GC**ACGGTTT**TTGC**A	797422	+	b0766	*ybhA*	-	b0767	*pgl*	368	10.051
IA10	1098490	6	A**TGGC**TTATTATA**TGC**A	1098486	-	b1034	*ycdX*	+	b1032	*serX*	1592	8.304
IA11	1272586	2	TC**GG**TACAGGTT**TTGC**A	1272583	+	b1219	*ychN*	-	b1220	*ychO*	405	10.68
IA12	1628490	2	C**TGG**TGGGGATT**TTGC**A	1628484	+	b1542	*ydfI*	-	b1544	*ydfK*	2593	10.373
IA13	1693966	2	C**TGGC**ACAGCAA**TTGC**C	1693959	+	b1617	*uidA*	-	b1621	*malX*	3401	11.352
IA14	1724329	2	C**TGG**TTCAGTGT**TTGC**T	1724326	-	b1649	*nemR*	+	b1648	*ydhL*	363	8.27
IA15	1781364	6	GC**GGC**ACGGAAAC**TGC**A	1781359	-	b1701	*fadK*	+	b1696	*ydiP*	4015	10.191
IA16	2060666	1	C**TGG**TCGATAAT**TTGC**A	2060657	+	b1990	*erfK*	-	b4582	*yoeA*	5983	7.431
IA17	2210856	1	CG**GGC**GCAGTTTA**TGC**A	2210849	+	b2125	*yehT*	-	b2127	*mlrA*	2020	9.868
IA18	2531372	8	C**TGGC**ATTACTG**TTGC**A	2531400	-	b2414	*cysK*	+	b2412	*zipA*	2126	11.499
IA19	2584411	3	AC**GG**TACAATTTA**TGC**A	2584425	-	b2469	*narQ*	+	b2468	*aegA*	859	10.049
IA20	2730510	1	A**TGG**TGCAGTTC**TTGC**T	2730500	+	b2592	*clpB*	-	b2595	*bamD*	3649	10.068
IA21	2795717	2	G**TGG**AATATAAT**TTGC**T	2795722	-	b2668	*ygaP*	+	b2666	*yqaE*	653	9.254
IA22	2960070	1	C**TGG**AACAGTTT**T**C**GC**T	2960081	+	b2822	*recC*	-	b2831	*mutH*	7584	9.118
IA23	3089038	1	C**TGGC**AAGCGCG**TTGC**A	3089053	-	b2945	*endA*	+	b2939	*yqgB*	4961	8.273
IA24	3110785	1	C**TGGC**TGATTAA**TTGC**A	3110786	+	b2970	*yghF*	-	b2980	*glcC*	15489	8.093
IA25	3152925	1	G**TGGC**ATAGGTT**T**C**GC**A	3152920	+	b3010	*yqhC*	-	b3011	*yqhD*	438	9.586
IA26	3440661	1	T**TGGC**GCTGTTTA**TGC**T	3440654	+	b3299	*rpmJ*	-	b3324	*gspC*	12927	10.636
IA27	3851258	2	TC**GGC**ACGAATT**TTG**AC	3851261	+	b4616	*istR*	-	b4618	*tisB*	296	8.863

^a^ Unique ID; IS = Inside a gene in the Sense orientation, IA = Inside a gene in the Antisense orientation; each peak is assigned a unique number for cross-referencing with other datasets

^b^ Underlined text indicates that a σ^54^ has been identified in the homologous position in *Salmonella* Typhimurium [14]

^c^ Genome coordinate (U00096.2) of ChIP-seq peak center

^d^ FAT = Fold Above Threshold score (indication of ChIP-seq occupancy)

^e^ Associated motif identified using MEME; consensus positions indicated in bold

^f^ Genome coordinate (U00096.2) of motif center

^g^ Genomic orientation of associated motif

^h^ Overlapping gene

^i^ Genomic orientation of overlapping gene

^j^ Closest, appropriately oriented, downstream gene (from the predicted transcription start site, 19 bp downstream of the motif center), excluding the gene that the binding site is within

^k^ Distance from the predicted transcription start site (19 bp downstream of the motif center) to the start of the closest, appropriately oriented, downstream gene (bp)

^l^ Position Specific Scoring Matrix (PSSM) score (indication of similarity to the consensus site; see Methods)

135 σ^54^ binding sites represents a very large increase over previously described, experimentally confirmed σ^54^ promoters, of which there are only 20 [26]. Nonetheless, we suspected that our stringent peak-calling algorithm may have missed some genuine σ^54^ promoters. Consistent with this, we failed to call a peak upstream of *crl*, despite two independent reports of σ^54^ binding [27,28]. Visual analysis of the ChIP-seq data for the region upstream of *crl* indicated a small peak, below the threshold used for peak calling (S2 Fig). We reduced the stringency of our peak-calling algorithm such that it identified an additional 204 “low stringency” peaks (i.e. 349 total peaks), including the site upstream of *crl*. We then analyzed the sequences surrounding these low stringency peaks using MEME [22]. We identified a highly enriched motif that closely resembles the known σ^54^–24/-12 promoter elements (S3A Fig). This motif is present in 149 of the 204 sequences (S2 Table) and is centrally enriched (*p* = 9.6e^-41^; S3B Fig). This is a smaller fraction than for the 135 sites identified using the stringent cut-off, indicative of a higher false positive rate. Nevertheless, it suggests that σ^54^ binds more than 250 sites in the *E*. *coli* genome.

### Comparison to previously described σ^54^ promoters

A recent study using ChIP-chip to map the binding of σ^54^ across the *E*. *coli* genome identified 161 putative σ^54^ binding sites [29]. We compared putative σ^54^ binding sites identified in the ChIP-chip study to the 135 high stringency σ^54^ binding sites we identified, in addition to the 10 sites we identified by ChIP-seq but which lacked a detectable motif. Strikingly, only 67 putative σ^54^ binding sites overlapped with our own list (S3 Table). We searched for enriched sequence motifs in the remaining 94 putative sites that were unique to the ChIP-chip study. The three most significantly enriched motifs identified by MEME [22] are all repetitive sequences from insertion elements (S4A Fig) and are not centrally enriched. There were no significantly enriched motifs that resembled the known σ^54^ promoter elements [6]. We also used MEME to search for enriched sequence motifs in the putative σ^54^ binding sites that were unique to our high stringency list. MEME detected a highly enriched motif that resembles the known σ^54^ promoter elements (MEME E-value = 5.7e^-83^; S4B Fig) [6]. This motif is centrally enriched (*p* = 4.8e^-28^; S4C Fig). We conclude that the majority of the σ^54^ binding sites identified using ChIP-chip are false positives and that many genuine sites were missed.

We compared our high stringency list of 135 σ^54^ binding sites to those identified in other studies. The EcoCyc database [26] lists 20 σ^54^ promoters that have been experimentally confirmed. We identified 18 of these (Table 1). Thus, our approach validates almost all known σ^54^ promoters. For one of the “known” σ^54^ promoters that we failed to identify, that for *ibpB*, we detected background levels of σ^54^ ChIP signal, suggesting that this is not a genuine σ^54^ promoter and is misannotated. The EcoCyc database [26] also lists 76 predicted σ^54^ promoters that have not been experimentally confirmed. These promoters have been predicted based on either DNA sequence or expression microarray data. We confirmed 15 of these predicted sites (Table 1).

A previous study used microarrays to compare expression of cells lacking σ^54^ to cells transiently overexpressing σ^54^ [28]. They identified 22 putative, novel σ^54^ promoters with high confidence. We identified appropriately orientated ChIP-seq peaks ≤500 bp upstream of only five of these genes (*norV*, *xdhA*, *ygfK*, *rutA* and *ddpX*). We used MEME [22] to search for enriched motifs in the regions upstream of the 22 putative, novel σ^54^-transcribed genes identified by [28] (including the five confirmed by our data). There were no significantly enriched motifs that resembled the known σ^54^ promoter elements (S5 Fig) [6]. We conclude that the majority of the σ^54^ binding sites identified using microarray analysis of RNA levels are false positives and that many genuine sites were missed.

### σ^54^ binding is associated with diverse gene functions

We selected all genes for which there was a σ^54^ binding site positioned <300 bp upstream in the same orientation as the gene. We then searched for enriched Gene Ontology (GO) terms using FuncAssociate [30]. We failed to identify any significantly enriched GO terms (*p* < 0.05 with multiple hypothesis testing correction), indicating that σ^54^-transcribed genes are not, as a group, strongly associated with any specific functions. This is consistent with previous studies that have identified a wide variety of functions for σ^54^-transcribed genes [6].

### Widespread intragenic σ^54^ binding with a non-random distribution

**Fig 3:**
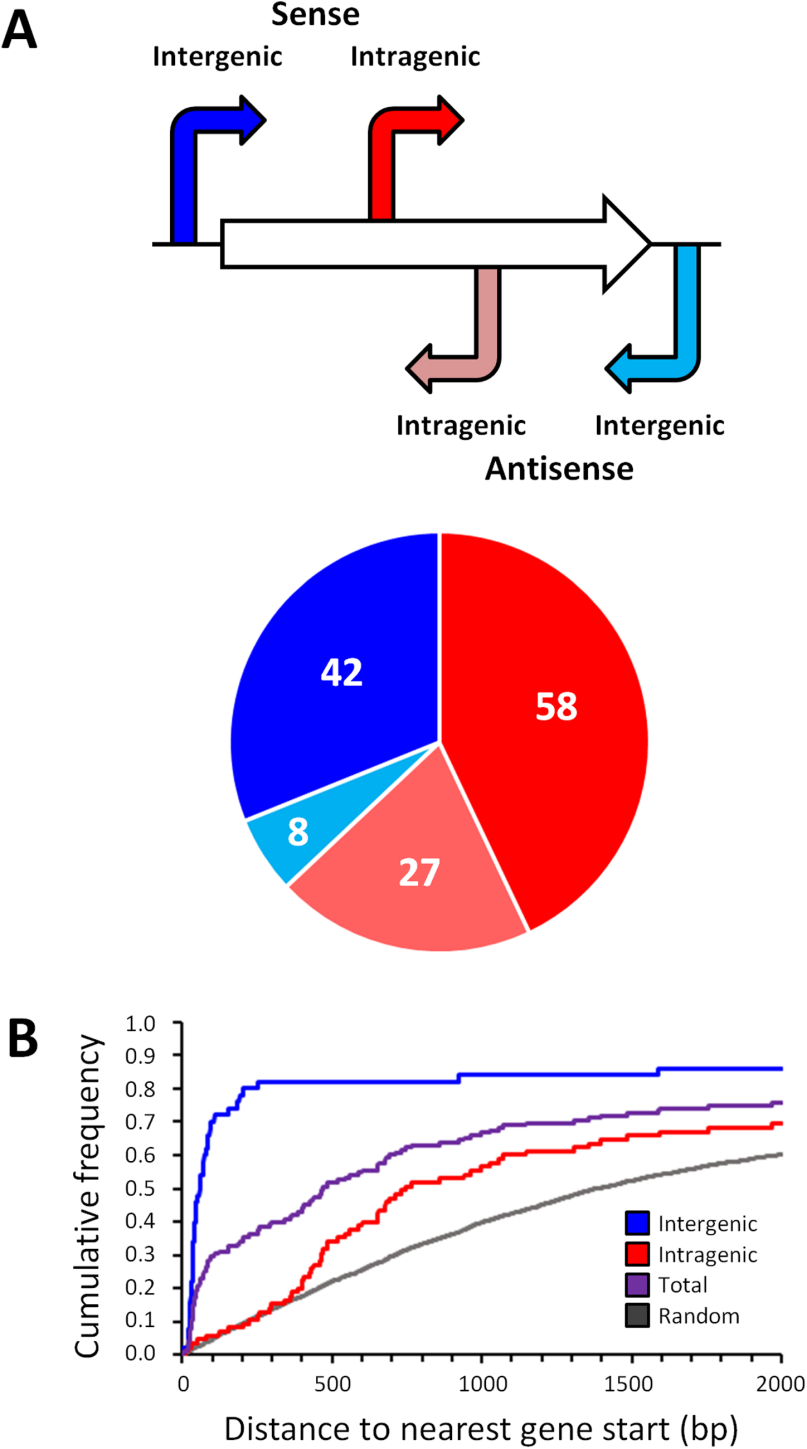
Distribution of σ^54^ binding sites relative to gene position. **(A)** (Top) Schematic representing the four possible classes of σ^54^ binding site relative to a gene. (Bottom) The distribution of each class of σ^54^ binding site in *E*. *coli*. **(B)** Cumulative frequency of the distance from intergenic (blue), intragenic (red) and all (purple) σ^54^ binding sites to the next available gene start. The cumulative frequency distribution of the distances between 4000 random positions and the next available gene start is also indicated (grey).

With one exception [20], all previously described, experimentally confirmed σ^54^ binding sites in *E*. *coli* are located in intergenic regions. The distribution with respect to gene position of the 135 high stringency σ^54^ binding site locations we identified is shown in Fig 3A. As expected, σ^54^ binding sites as a group are closer to annotated gene starts than random genomic positions (Fig 3B). Surprisingly, 85 of the 135 σ^54^ binding sites (62%) are located inside genes (Fig 3A). Furthermore, of the 50 σ^54^ binding sites in intergenic regions, 8 are oriented away from the neighboring gene(s), indicating that they are not promoters for the immediately adjacent genes. Further analysis of the σ^54^ binding sites inside genes showed that 58 (68%) of these sites are positioned in the sense orientation with respect to the overlapping gene (Fig 3A). This is far more than expected by chance (Binomial test *p* = 5e^-4^, assuming a 50% random chance of sense/antisense orientation). We observed a similar, significant bias (79/129; 61%) for the low stringency sites (Binomial test *p* = 0.007; S2 Table). The distance of intragenic σ^54^ binding sites from the nearest appropriately oriented gene start is also non-random. Specifically, there are many more sites between 360 and 760 bp upstream of an available gene start than expected by chance (36.5% of all intragenic sites as compared to 14.3% of all intragenic coordinates genome-wide; Fig 3B).

### σ^54^ typically binds in the context of transcriptionally inactive RNAP holoenzyme

**Fig 4:**
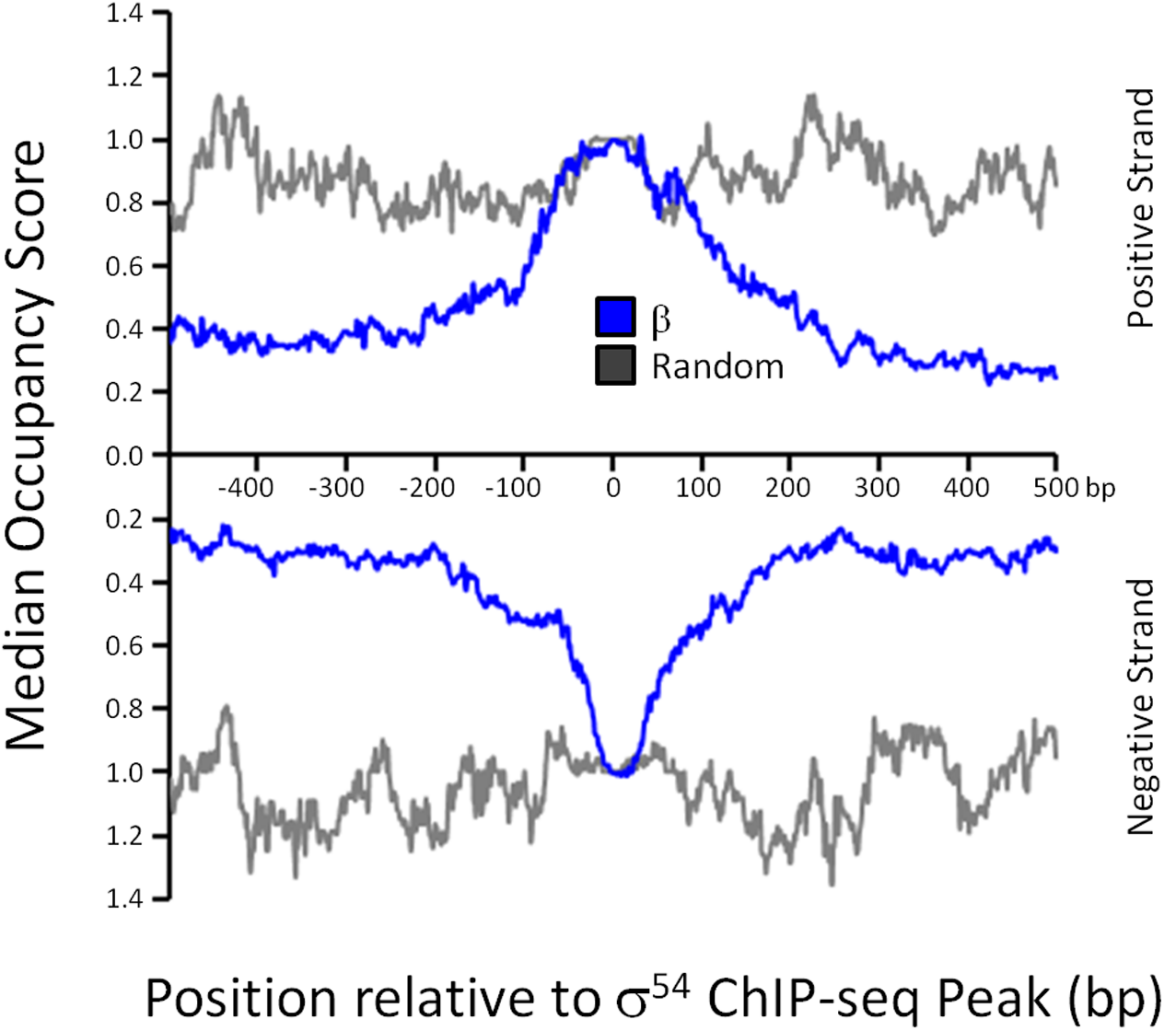
RNAP distribution at σ^54^ binding sites. The median RNAP (β) occupancy (median occupancy score) was determined using ChIP-seq for positions from -500 to +500 bp relative to each σ^54^ ChIP-seq peak. These data are shown in blue, with separate lines for each strand. The orientation was determined based on the identified σ^54^ binding motif. All binding sites are oriented for potential transcription in the downstream direction. Data shown in grey are for an equivalent control analysis using 135 randomly selected genomic positions. Values on the *x*-axis indicate position relative to σ^54^ ChIP-seq peaks (bp).

**Fig 5:**
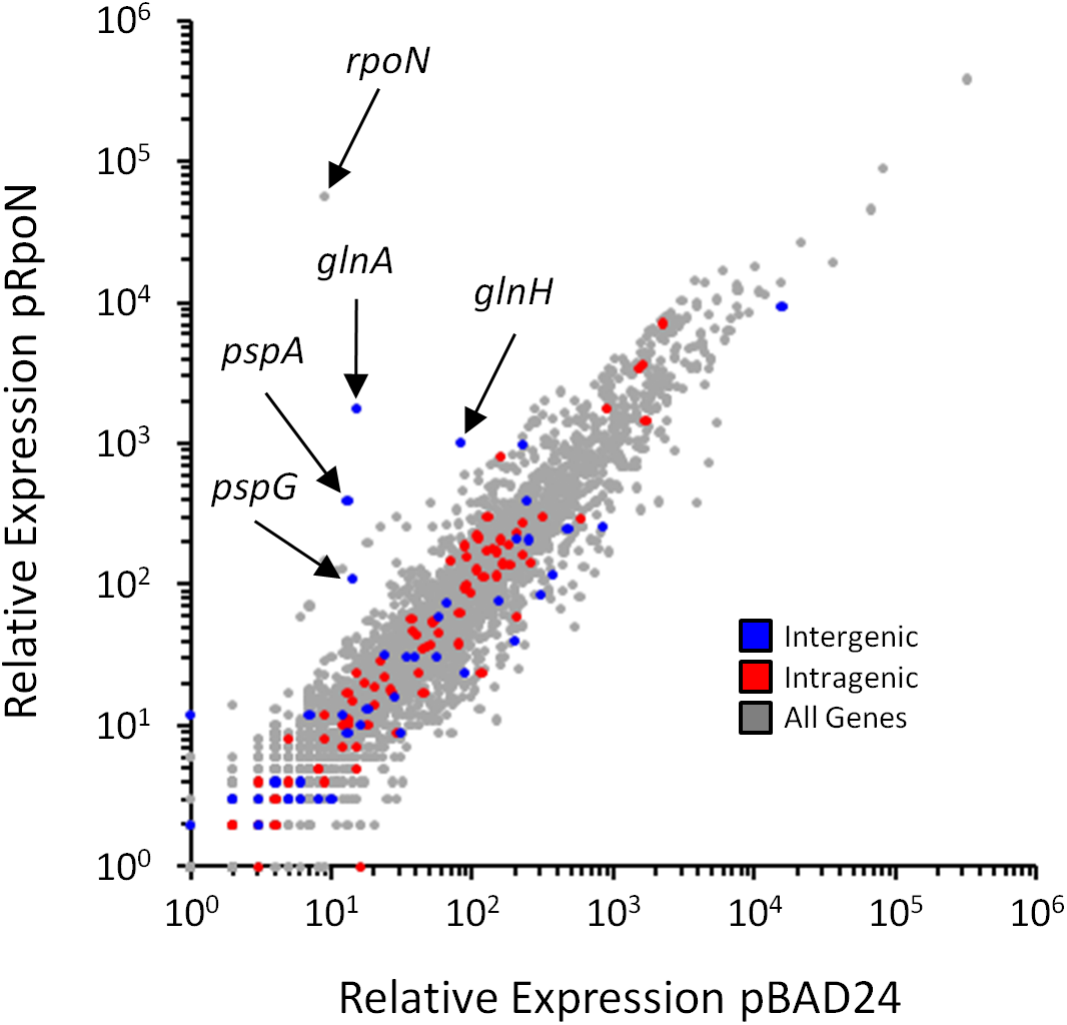
Genome-wide σ^54^-dependent changes in gene expression. Relative RNA levels, determined by RNA-seq, for all genes in cells transiently overexpressing *rpoN* (MG1655 *ΔrpoN* + pRpoN; RPB149) or control cells containing empty vector (MG1655 *ΔrpoN* + pBAD24; RPB152). Relative RNA levels were calculated using Rockhopper [65]. Each gene is indicated by a grey data point. Genes immediately downstream of intergenic σ^54^ sites (blue), or genes containing intragenic σ^54^ sites (red) are highlighted.

σ^54^ can bind promoter DNA in the absence of core RNAP *in vitro*, although the affinity is lower than that in the context of RNAP holoenzyme [5]. To determine whether σ^54^ binding *in vivo* is in the context of RNAP holoenzyme, we used ChIP-seq to map the genome-wide distribution of the β subunit of core RNAP under the same growth conditions used for σ^54^ ChIP-seq. As shown in Fig 1A, we detected increased local signal for β at sites of σ^54^ binding. In some cases, especially for intragenic σ^54^ binding sites, the σ^54^ peak is located within a transcribed region. Hence, it is more difficult to ascertain σ^54^-dependent RNAP binding at these positions. Therefore, we determined the median occupancy of RNAP at each of the positions in the 1 kbp region surrounding all σ^54^ binding sites (Fig 4; note that each σ^54^ peak contributes equally in this analysis, regardless of the ChIP-seq signal for σ^54^). RNAP binding is substantially greater at the exact site of σ^54^ binding than in the flanking sequence. Furthermore, RNAP binding at the motif center is enriched compared to RNAP binding at randomly selected positions throughout the genome (Fig 4). We conclude that most or all σ^54^ binding *in vivo* is in the context of RNAP holoenzyme. We also noted that the distribution of RNAP binding at σ^54^ binding sites is symmetric (Figs 1A and 4), indicative of RNAP that is not actively transcribing RNA. Thus, our data strongly suggest that most or all RNAP:σ^54^ is transcriptionally inactive under the conditions tested. Furthermore, as described in more detail below, little RNA initiates from identified σ^54^ binding sites under the conditions used in our study (Fig 5 and S4 Table).

### σ^54^ binding to intragenic sites does not impact expression of the overlapping genes

It is intriguing that (i) a large proportion of σ^54^ binding sites is intragenic (Fig 3A), (ii) 52% of σ^54^ binding sites are >500 bp from an annotated gene start (Fig 3B), (iii) σ^54^ binding at most sites appears to be transcriptionally inactive (Fig 4), and (iv) σ^54^ has been previously shown to repress transcription when its binding site overlaps another promoter [27,31]. Based on these observations, we postulated that intragenic σ^54^ binding may reduce expression of the overlapping genes by acting as a transcriptional “roadblock” [32]. To test this hypothesis, we compared the expression of all genes using RNA-seq in cells lacking σ^54^ and cells transiently overexpressing σ^54^. We observed significant (≥ 2-fold regulation with an estimated False Discovery Rate ≤ 0.01) expression differences for 465 genes. 272 genes were up-regulated following σ^54^ overexpression, while 193 were down-regulated (Fig 5 and S4 Table). Of the 85 genes with intragenic σ^54^ binding sites, only *ybaT*, *erfK* and *clpB* were significantly down-regulated (S4 Table). Down-regulation of three out of 85 genes is not significantly more than expected by chance given the total number of down-regulated genes (Fisher’s exact test *p* = 0.47). Therefore, it is unlikely that RNAP:σ^54^ acts as a transcriptional roadblock under these conditions.

We also examined the genes that were up-regulated upon σ^54^ overexpression. Only 5 genes with intergenic σ^54^ binding sites upstream were significantly up-regulated. These genes are all known to be transcribed by RNAP:σ^54^. For each gene, regulation by a known bEBP has been described previously: *glnA* and *glnH* by NtrC [33], *pspA* and *pspG* by PspF [34] and *glmY* by GlrR (Fig 5) [35]. No other known RNAP:σ^54^-transcribed genes were significantly up-regulated. Ten genes with intragenic σ^54^ sites were significantly up-regulated following σ^54^ overexpression. Up-regulated genes *carB*, *purK*, *wbbK*, *yhbE* and *rplD* contain a σ^54^ motif in the sense orientation relative to the overlapping gene. However, genes immediately upstream were also up-regulated, suggesting an indirect effect of σ^54^ overexpression on whole operons. *rpmJ*, *metQ*, *yajG* and *cysK* were also up-regulated by σ^54^ overexpression. However, the motif associated with these binding sites is in the antisense orientation. Finally, *metF*, a gene up-regulated upon σ^54^ overexpression, contains an intragenic σ^54^ binding site in the sense orientation that is not associated with up-regulation of an upstream gene. However, the σ^54^ site is located near the 3ʹ-end of the gene, suggesting that it is not responsible for the observed increase in expression. We conclude that up-regulation of genes that contain intragenic σ^54^ binding sites is not due to binding of σ^54^ at these positions, but rather to indirect effects of σ^54^ overexpression.

### Some intragenic σ^54^ binding sites are functional promoters

**Fig 6:**
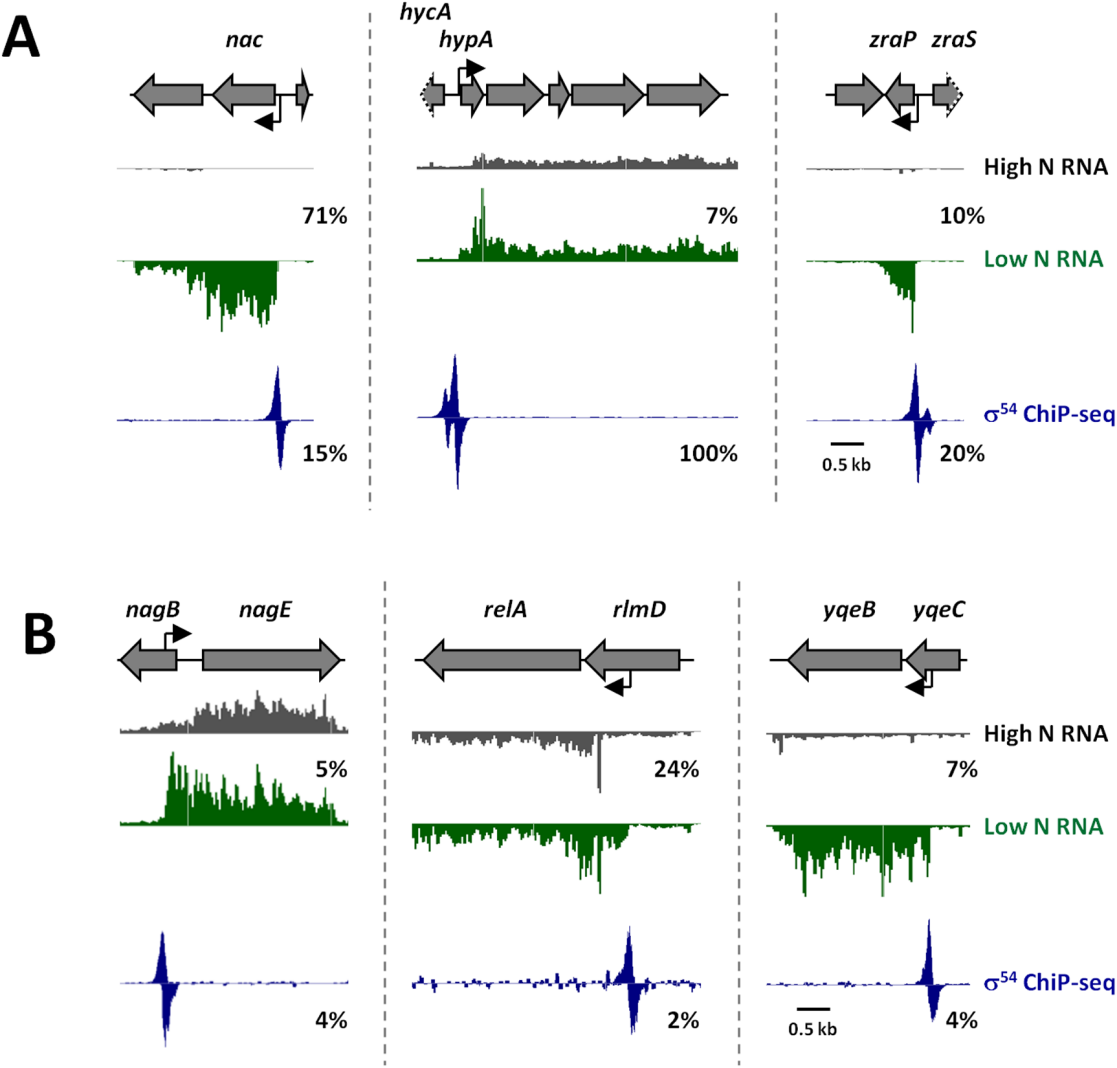
Examples of novel σ^54^ promoters associated with transcription under conditions of nitrogen limitation. Microarray data from [36] showing RNA levels at selected regions around novel **(A)** intergenic or **(B)** intragenic σ^54^ promoters. The σ^54^ promoter upstream of *nac* has been previously described [71] and serves as a positive control. Grey arrows represent genes. Grey arrows with dotted lines indicate that only a portion of the gene is shown. Bent, black arrows represent σ^54^ promoter motifs. Grey and green histograms indicate RNA levels in nitrogen-rich and nitrogen-limiting media, respectively and have been scaled equivalently. Blue histogram indicates σ^54^ ChIP-seq occupancy. Percentages indicate relative scale on the y-axis.

Our RNA-seq analysis indicates that only three bEBP activators, NtrC, PspF and GlrR, are active under the conditions used, most likely at a very low level (Fig 5 and S4 Table). A single condition where most bEBPs are induced has not been determined; hence, most σ^54^ promoters will be inactive under a given condition. The bEBP NtrC is highly active in nitrogen-limiting conditions [20]. We compared our ChIP-seq data to published microarray data from *E*. *coli* grown under nitrogen-rich conditions (NtrC inactive), and conditions known to induce activity of NtrC [36]. Not all of the σ^54^ initiated genes are expected to be up-regulated under these conditions, just those under control of NtrC. There are 15 previously described NtrC-regulated operons in *E*. *coli* [20,37]. All of these operons are associated with a σ^54^ ChIP-seq peak (our study), and most are associated with observed increases in expression in nitrogen-limiting conditions, e.g. *nac* (Fig 6A) [29]. We observed two additional intergenic ChIP-seq peaks associated with increased expression of the downstream genes, *hypA* and *zraP*, in nitrogen-limiting conditions (Fig 6A). *hypA* and *zraP* are both known to be transcribed by RNAP:σ^54^; however, transcription of these genes is activated by bEBPs other than NtrC. Specifically, transcription of *hypA* is activated by FhlA in response to anaerobiosis and the presence of formate [38], and transcription of *zraP* is activated by ZraR in response to high zinc concentrations [39]. Thus, our data suggest either that *hypA* and *zraP* are regulated by multiple bEBPs (i.e. NtrC and at least one other), or that bEBPs other than NtrC are activated under the growth conditions tested. The latter is more likely since no NtrC binding was detected upstream of *hypA* or *zraP* by ChIP-seq [20]. We also observed three transcripts induced under low nitrogen conditions that initiate from intragenic σ^54^ binding sites. These σ^54^ binding sites, within *nagB*, *yqeC* and *rlmD*, are located immediately upstream of the start sites for the transcripts induced by nitrogen limitation, and the predicted -24 and -12 motifs are oriented in the same direction as the associated RNAs (Fig 6B). In each case, the RNA that appears to be transcribed by RNAP:σ^54^ extends through the adjacent gene (Fig 6B). Thus, these RNAs appear to be mRNAs with unusually long 5ʹ UTRs. A recent study identified an NtrC-activated σ^54^ promoter inside *rlmD* [20]. This promoter is an exact match to the one we identified, and drives transcription of the entire *relA* gene (adjacent to *rlmD*), consistent with our analysis.

**Fig 7:**
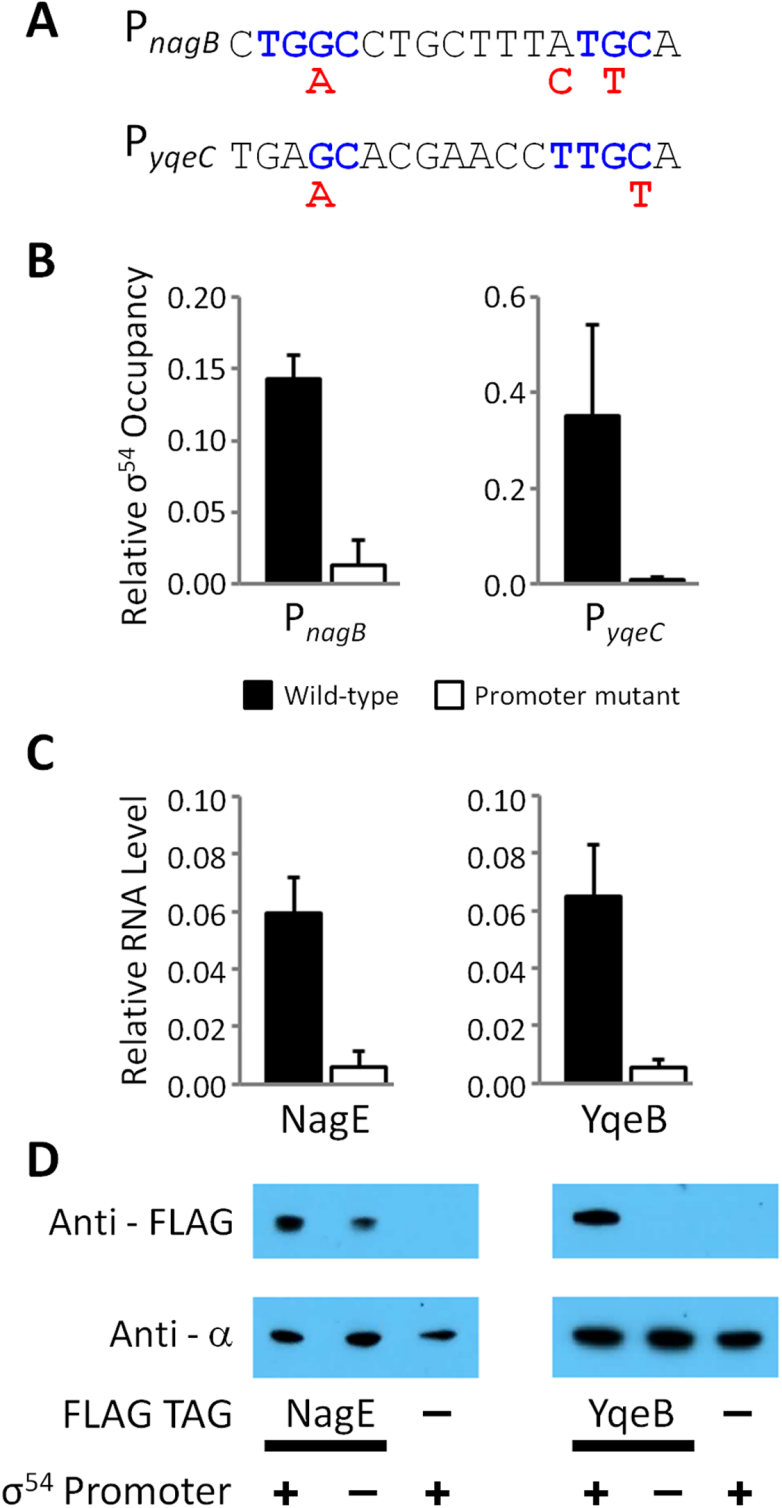
Validation of intragenic σ^54^ promoters for the *nagE* and *yqeB* mRNAs. **(A)** Intragenic P_*nagB*_ and P_*yqeC*_ σ^54^ promoters were chromosomally mutated. The sequence of both σ^54^ promoters is shown; conserved, consensus residues (blue text) and the mutagenic changes (red text) are indicated. **(B)** The relative σ^54^ occupancy compared to a positive control region (the σ^54^ promoter of *glnA*) was measured by ChIP-qPCR at both promoters in wild-type (RPB220 for *nagB* and RPB232 for *yqeB*; black bars), ΔP_*nagB*_ (RPB277; white bars) and ΔP_*yqeC*_ (RPB279; white bars) *E*. *coli* strains. Note that “wild-type” and “mutant” refer to the status of the promoter. Strains used to evaluate P_*nagB*_ and NagE contain a C-terminal FLAG-tagged *nagE* gene, whereas the strains used to evaluate P_*yqeC*_ and YqeB contain a C-terminal FLAG-tagged *yqeB* gene. **(C)** Expression of *nagE* and *yqeB*, the genes immediately downstream of P_*nagB*_ and P_*yqeC*_ σ^54^ promoters, respectively, relative to the expression of *glnA*, was measured by RT-qPCR. **(D)** Western blot probing of extracts from NagE and YqeB FLAG-tagged and untagged *E*. *coli* strains with anti-FLAG antibody. Probing the same membranes with anti-α (RNAP subunit) antibody served as a loading control. Wild-type (+) and mutated (-) promoters are indicated. The blot is representative of three independent biological replicates.

To further investigate the intragenic σ^54^ binding sites within *nagB* and *yqeC*, we constructed strains with epitope tags fused to 3ʹ ends of *nagE* or *yqeB*. We then constructed derivatives of these strains with mutations in the σ^54^ binding site inside *nagB*, or *yqeC*, respectively (one silent change and one His → Lys codon change in *nagB*; two silent changes in *yqeC*; Fig 7A). We measured association of σ^54^ with the wild type and mutated sites using ChIP-qPCR. Our data indicate that mutating the putative binding sites greatly reduces binding of σ^54^, confirming that these are genuine σ^54^ binding sites (Fig 7B). The microarray data described above strongly suggested that each of these intragenic σ^54^ binding sites is a promoter for an mRNA for the downstream gene. To test this hypothesis, we used qRT-PCR to measure mRNA levels of the downstream gene for each putative promoter (*nagE* and *yqeB*) in wild type cells and cells in which the binding site is disrupted (Fig 7C). These data indicate that mutation of either σ^54^ binding site results in a large decrease in the mRNA level for the downstream gene. Lastly, we used Western blotting with an antibody specific to the epitope tags to measure NagE and YqeB protein levels in cells with wild type and mutant promoters. Consistent with the qRT-PCR data, mutation of either promoter resulted in a decrease in the protein level for the downstream gene (Fig 7D). In the case of NagE, the decrease in protein level was modest (~2-fold), whereas YqeB was undetectable in the promoter mutant. We conclude that the σ^54^ binding sites within *nagB* and *yqeC* represent promoters for *nagE* and *yqeB*, respectively, with the mRNAs having unusually long 5ʹ UTRs.

### Conservation of σ^54^ binding sites suggests that many intragenic σ^54^ binding sites are functional

**Fig 8:**
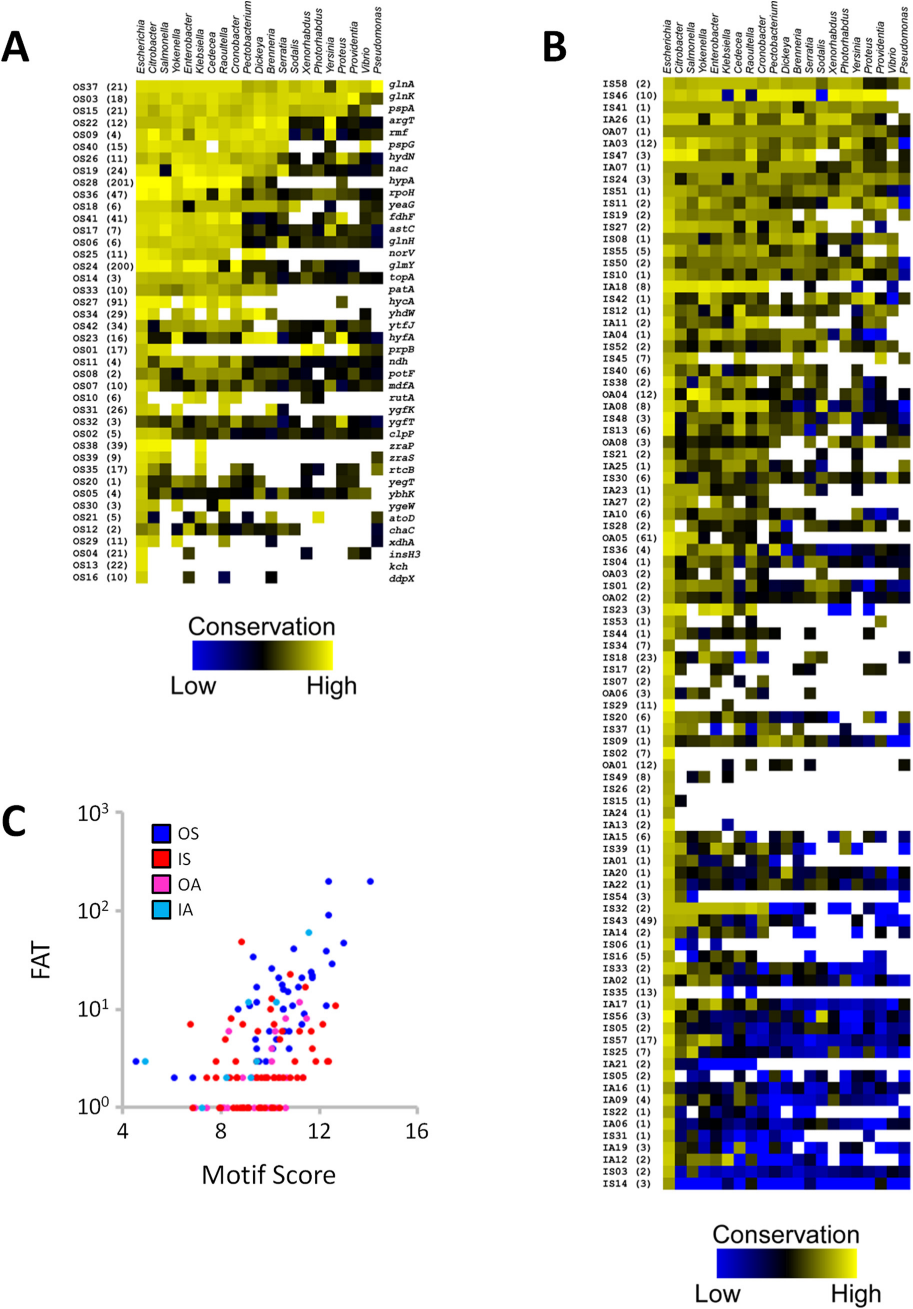
Conservation analysis of σ^54^ binding sites. Heat-maps depicting the match to the σ^54^ consensus binding site for each **(A)** canonical and **(B)** non-canonical σ^54^ binding site across a range of bacterial species. Genera are listed across the top, binding site ID numbers, and fold above threshold (FAT) scores in parentheses are listed to the left of the heat-map. For σ^54^ binding sites in panel A, the gene immediately downstream of each binding site is indicated to the right of the heat-map. **(C)** Comparison of the level of σ^54^ binding, as indicated by FAT score (Tables 1 & 2), versus Motif Score (S5 Table) for *E*. *coli* only. Different classes of binding site are indicated by color.

We speculated that other intragenic σ^54^ binding sites represent genuine promoters. We therefore compared each site across a range of bacterial species, mostly from the family *Enterobacteriaceae*. σ^54^ is well conserved across these species (e.g. 62% identical, 79% similar amino acid sequence between *E*. *coli* and *V*. *cholerae*), suggesting that it binds with similar DNA sequence specificity. Furthermore, ChIP-seq of σ^54^ in *V*. *cholerae* identified a very similar motif to the *E*. *coli* σ^54^ motif identified here (Fig 1B) [19], despite the two species having diverged >600 million years ago [40]. Therefore, we used a position weight matrix derived from our MEME analysis (Fig 1B) to score sequences, from other species, that correspond to homologous regions to those surrounding the 135 high stringency σ^54^ binding sites in *E*. *coli*. In some cases, no homologous region was identified. A summary of the conservation analysis is shown in Fig 8, and a complete list of conservation scores is shown in S5 Table. Our analysis indicated that, as a group, intergenic binding sites (Fig 8A) are better conserved than intragenic sites (Fig 8B). However, many intragenic σ^54^ binding sites are conserved, suggesting that these sites are functional. Importantly, the computationally determined motif score correlates well with *in vivo* binding of σ^54^ (Fig 8C): the Spearman’s Rank Correlation Coefficient for the “OS” class of binding site (intergenic, oriented towards a gene) is 0.67, and for the rest of the binding sites is 0.38.

### Functional conservation of intragenic σ^54^ binding sites in *Salmonella* Typhimurium

**Fig 9:**
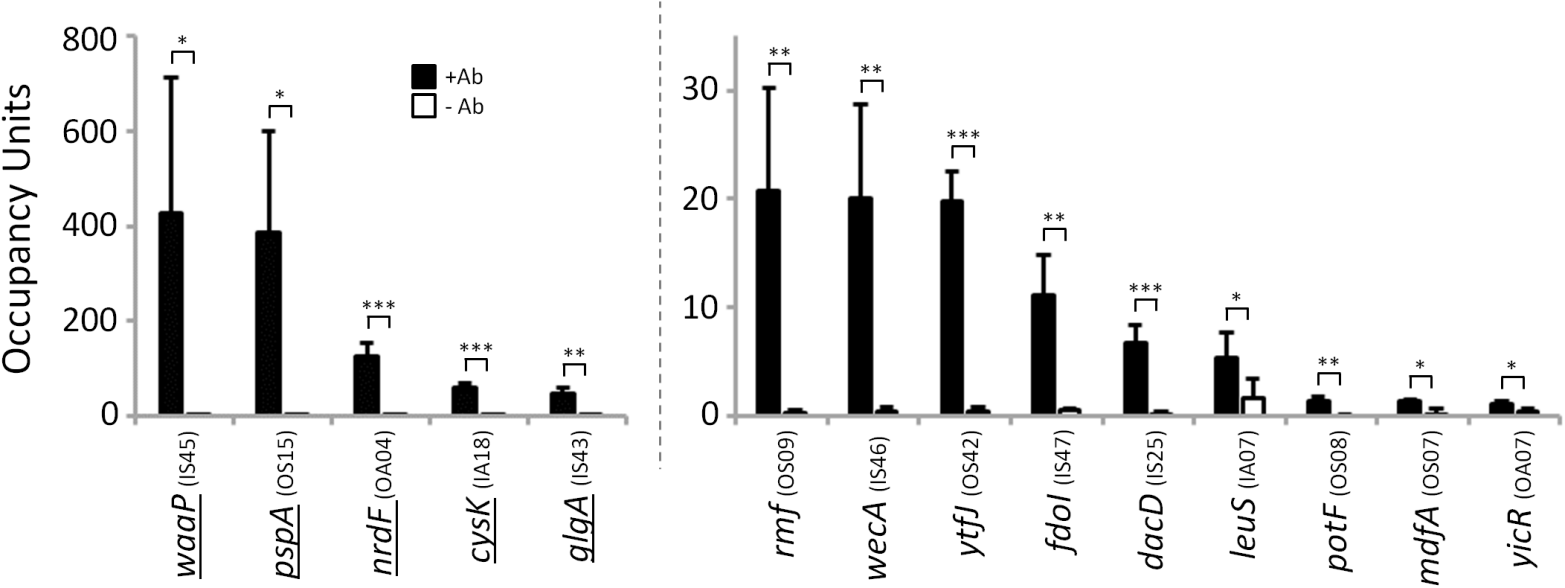
σ^54^ binds conserved sites in *Salmonella enterica*. Targeted validation of predicted σ^54^ binding sites in *S*. *enterica*. Enrichment (occupancy units) at predicted σ^54^ binding sites in *S*. *enterica* was measured by ChIP-qPCR with anti-σ^54^ (black bars) or no antibody (white bars). The cognate promoter IDs (Tables 1 & 2) are indicated in parentheses. Error bars represent the standard deviation from three independent biological replicates. Significant differences between wild-type and Δ*rpoN* values are indicated (*p ≤ 0.05, **p ≤ 0.01, ***p ≤ 0.001). Note that the *y*-axis scale differs for first five regions indicated. Underlined genes indicate σ^54^ binding sites previously identified by [14].

The sequence-based phylogenetic analysis described above predicted that many of the σ^54^ sites we detected in *E*. *coli* are functionally conserved in *Salmonella enterica*. To test this prediction, we used ChIP-qPCR to measure association of σ^54^ with 14 sites in *Salmonella enterica* serovar Typhimurium that were predicted on the basis of sequence conservation. Only five of these sites are in the classical “outside sense” orientation (intergenic, oriented towards a gene). As a control, we performed ChIP-qPCR without antibody. In all cases, we detected robust association of σ^54^, dependent upon the presence of antibody in the ChIP (Fig 9). We conclude that many intragenic σ^54^ binding sites are functionally conserved in *S*. *enterica*. Moreover, these data validate the sequence-based predictions of conservation (Fig 8).

## Discussion

### A greatly expanded set of σ^54^ binding sites

Unlike members of the σ^70^ family, σ^54^ proteins can bind promoters, in the context of RNAP holoenzyme, in a transcriptionally inactive state. Therefore, while σ^54^-dependent transcription will only occur under conditions that activate the relevant bEBPs, the binding of σ^54^:RNAP holoenzyme to promoter DNA is not condition-dependent. Our work takes full advantage of this characteristic to map the σ^54^ promoters of the *E*. *coli* genome using ChIP-seq. In contrast, we reasoned that genome-wide expression levels of most σ^54^-transcribed genes are unlikely to differ in cells lacking σ^54^, since most bEBPs are inactive in standard growth media. This is supported by our RNA-seq analysis, which identified only 5 σ^54^-transcribed RNAs (Fig 5 and S4 Table), and by a genome-scale analysis of σ^54^ in *V*. *cholerae* [19]. Only 20 σ^54^ promoters have previously been experimentally validated in *E*. *coli* [26]. Using ChIP-seq, we have confirmed 19 of these and have vastly expanded the list of known σ^54^ binding sites to 135. Moreover, our ChIP-seq data analysis with a relaxed cut-off is consistent with >250 σ^54^ binding sites, based on those for which we identified a motif. Our data indicate that, for the conditions we used, σ^54^ binds most or all sites in the context of transcriptionally inactive RNAP holoenzyme (Figs 4 and 5). As discussed below, many of the novel sites are likely to be non-functional, but there is strong evidence that a substantial subset has conserved function.

In addition to the 19 previously described σ^54^ promoters verified by our work, we confirmed 15 predicted σ^54^ promoters. Our data are inconsistent with most of the previous predicted σ^54^ promoters [26]; false negatives due to the conditions we used are possible, but unlikely since σ^54^ is expected to bind all DNA sites under all growth conditions in which σ^54^ is expressed. We have also identified 8 novel σ^54^ binding sites in intergenic regions, close to an appropriately oriented gene (Table 1). This brings the total of canonical σ^54^ binding sites, i.e. those that are intergenic and likely to represent promoters for the downstream gene, to 42. The identities of these genes support a role for σ^54^ in transcribing genes involved in a wide range of cellular functions.

### Extensive intragenic σ^54^ binding

The majority of the 135 high stringency σ^54^ binding sites we identified are located inside genes. Moreover, eight of the intergenic σ^54^ binding sites are located far (>300 bp) from the nearest appropriately oriented gene (Fig 3A). A recent ChIP-chip study of σ^54^ in *Salmonella enterica* also suggested the existence of intragenic binding sites, although the resolution of that study was at the level of whole genes due to the microarray design (PCR products for entire genes) [14]. A ChIP-seq study of σ^54^ in *V*. *cholerae* identified only 7 intragenic σ^54^ binding sites [19], suggesting that the function of σ^54^ in some species may be more restricted. This is consistent with our analysis of σ^54^ binding site conservation, which showed conservation of very few binding sites between *E*. *coli* and *V*. *cholerae* (Fig 8).

Only a few other alternative σ factors have been mapped using ChIP-chip or ChIP-seq [10,11,13,15,16,17,41,42], and in most cases the large majority of reported binding sites were intergenic. However, *E*. *coli* σ^32^, σ^28^, and *Mycobacterium tuberculosis* σ^F^ were reported to bind large numbers of intragenic sites [15,16,17,18]. In all three cases, a few intragenic binding sites were associated with transcription of RNAs that initiate inside a gene, sometimes in the antisense orientation [15,16,18].

### Conservation of many intragenic σ^54^ binding sites suggests extensive biological function

Intragenic σ^54^ binding sites as a group have significantly lower σ^54^ occupancy, as measured by ChIP-seq, than intergenic binding sites (Mann Whitney U Test *p* < 1e^-8^). This suggests that many intragenic σ^54^ binding sites represent “biological noise”, binding sites created by chance due to sequence constraints imposed by other factors such as coding sequence. However, our data indicate that σ^54^ binding site function and the level of σ^54^ binding do not correlate well. For example, σ^54^ binding sites within *nagB*, *rlmD* and *yqeC* are associated with transcription of mRNAs (Figs 6 and 7) but have relatively low binding scores (Fold Above Threshold scores (see Methods) of 8, 2 and 7, respectively).

Our bioinformatic analysis indicates widespread sequence conservation of many intragenic σ^54^ binding sites (Fig 8), strongly suggesting important biological functions for these sites. In some cases, conservation is restricted to a few species. Nonetheless, even conservation solely in *S*. *enterica*, one of the closest relatives to *E*. *coli* that we analyzed, is a strong indication of function, since *E*. *coli* and *S*. *enterica* diverged from a common ancestor ~100 million years ago [43]. We compared the σ^54^ binding sites identified in our study to those identified by ChIP-chip in *S*. *enterica* [14]. Some predicted σ^54^ binding sites in *S*. *enterica* are shared with those in *E*. *coli*. In particular, 20 intergenic σ^54^ binding sites are common to both species (Table 1). Only six intragenic σ^54^ binding sites described in *S*. *enterica* correspond to genes for which we detected an intragenic site in *E*. *coli* (Table 2). However, this low degree of overlap is likely due to technical limitations of the ChIP-chip method on sensitivity and resolution. Indeed, many more of the σ^54^ binding sites that we identified in *E*. *coli* are conserved at the sequence level in *S*. *enterica* (Fig 8). Consistent with this, we detected association of σ^54^ with five such sites in *S*. *enterica* as well as four of the sites identified in the *S*. *enterica* ChIP-chip study (Fig 9). Thus, many σ^54^ binding sites are not only conserved between *E*. *coli* and *S*. *enterica* at the sequence level, but are also functionally conserved.

It is possible that conservation of some σ^54^ binding sites could be explained by sequence constraints imposed by the amino acid sequence of the overlapping protein, e.g. for the binding site within the highly conserved gene *rpmJ*. However, in many cases, the predicted level of σ^54^ binding is similar between species even though the DNA sequence changes. For example, changes can occur at positions that do not contribute to σ^54^ binding (e.g. position 9 in Fig 1B). To specifically address this question, we compared conservation of “important” bases within intragenic σ^54^ sites (i.e. those that rarely deviate from the consensus; positions 3, 4, 14, 15 and 16 in Fig 1B) to that of ‘unimportant” bases (i.e. those with low information content in the σ^54^ motif; positions 1, 8, 9, 10 and 12 in Fig 1B). If the σ^54^ binding site is the subject of positive selection, the unimportant bases should be less well conserved than the important bases, reflecting the sequence requirements for σ^54^ binding. In contrast, if selection is acting on the amino acid coding capacity in the region encompassing the σ^54^ binding site, the unimportant and important bases should be conserved to a similar degree. For each intragenic σ^54^ binding site in *E*. *coli* (Table 2), we selected all strong predicted sites (PSSM score >6; S5 Table) that are located at the equivalent position within homologous genes in other species. We then determined the number of times important base positions differed between *E*. *coli* and non-*E*. *coli* species (S6 Fig; note that we only analyzed positions for which the *E*. *coli* motif matched the consensus). Very few (35 out of a possible 1603) substitutions occurred at these important positions. This low frequency of substitution was expected since we selected only strong predicted σ^54^ sites. In contrast, we observed an 8.6-fold higher rate of substitution (309 out of a possible 1640) at unimportant positions (S6 Fig). We conclude that, in most cases, conservation of intragenic σ^54^ sites is limited to the important base positions, and that intragenic σ^54^ promoters are the target of positive selection rather than the surrounding protein-coding sequence.

### Many intragenic σ^54^ binding sites likely represent promoters for mRNAs

The conditions used in our RNA-seq analysis are not associated with the activity of most *E*. *coli* bEBPs (Fig 5). Hence, it is not possible to use these data to identify those σ^54^ binding sites that represent promoters. Nonetheless, our analysis of published microarray data for nitrogen-rich and nitrogen-limiting conditions suggested three σ^54^ binding sites inside *nagB*, *rlmD* and *yqeC*, as promoters for mRNAs for *nagE*, *relA* and *yqeB*, respectively (Fig 6). Targeted mutations in the σ^54^ binding sites inside *nagB* and *yqeC* confirmed they are genuine promoters (Fig 7). In each case, the promoter drives transcription of an mRNA for the downstream gene, and the 5ʹ UTR is unusually long. The third σ^54^ binding site, that within *rlmD*, was recently described by another group as a promoter for the downstream gene, *relA* [20]. This mRNA also has an unusually long 5ʹ UTR. The functional importance of the three intragenic σ^54^ promoters identified thus far in *E*. *coli* is emphasized by their conservation in other species (Fig 8) [14].

The functions of RelA and NagE are consistent with regulation of their respective genes by NtrC. Transcription of *relA* under nitrogen-limiting conditions couples nitrogen stress to the stringent response [20]. NagE helps to scavenge nitrogen by importing N-acetyl-D-glucosamine 6-phosphate, which can be metabolized by NagA and NagB to yield nitrogen in the form of ammonium [26]. In contrast, there is no obvious connection between nitrogen starvation and YqeB, a putative selenium-dependent molybdenum hydroxylase [44].

It is striking that there is a significant enrichment for intragenic σ^54^ binding sites that are in the sense orientation relative to the overlapping gene (Fig 3A). In most cases, this places them in the sense orientation relative to the downstream gene. Furthermore, there is an enrichment for intragenic σ^54^ binding sites 360–760 bp upstream of the downstream gene (Fig 3B). All three of the confirmed RNAP:σ^54^-transcribed mRNAs that initiate inside genes have 5ʹ UTRs between 360 and 760 nt long. Together, these observations strongly suggest that many intragenic σ^54^ binding sites represent promoters for the downstream gene, and the corresponding mRNAs include 5ʹ UTRs that are between 360 and 760 nt long.

What is the functional significance of σ^54^-transcribed mRNAs that have long 5ʹ UTRs? In the case of mRNAs that initiate antisense to other genes, the σ^54^-transcribed mRNAs or the act of their transcription could regulate expression of the overlapping gene. Indeed, mRNAs with long 5ʹ UTRs that are antisense to adjacent genes have been described in *Listeria monocytogenes*, and have been proposed to regulate expression of the overlapping genes [45], and regulation by antisense RNAs through base-pairing interactions or by transcriptional interference has been extensively described [46,47]. For mRNAs with long 5ʹ UTRs that initiate inside genes in the sense orientation, the functional significance is less clear. However, our data suggest that 5ʹ UTR length may be important for control of translational efficiency. In the case of *nagE*, mRNA levels were greatly reduced by mutation of the σ^54^ promoter in *nagB*, but protein levels decreased only ~2-fold (Fig 7). This is consistent with the presence of a second, σ^54^-independent *nagE* mRNA that is more efficiently translated. We propose that the long 5ʹ UTRs of σ^54^-transcribed mRNAs allow for more extensive control of translation efficiency. An alternative function for the long 5ʹ UTRs of σ^54^-transcribed mRNAs is as regulators: regulation by an mRNA 5ʹ UTR has been recently described for an mRNA in *Streptococcus mutans* [48].

### Other possible functions of intragenic σ^54^ binding sites

An alternative explanation for the overrepresentation of “sense” intragenic σ^54^ binding sites is that antisense binding sites interfere with transcription of the overlapping gene and are selected against. A recent study compared our list of σ^54^ binding sites with RNA-seq data for cells with or without *rpoN* [49]. This comparison suggested (i) competition between σ^54^ and other Sigma factors for binding to sites in intergenic regions, and (ii) regulation by RNAP:σ^54^ binding within genes. Our RNA-seq data are consistent with regulation by competition between Sigma factors at intergenic sites (Fig 5 and S4 Table), but we observed no effect of intragenic σ^54^ binding sites on expression of the overlapping gene (Fig 5 and S4 Table), effectively ruling out the possibility of roadblock repression. However, for the conditions used in our study, RNAP:σ^54^ bound at intragenic sites is not transcriptionally active (Fig 5). Hence, we cannot rule out transcriptional interference from active intragenic σ^54^ promoters.

Recent studies have identified many novel binding sites for transcription factors that are located far from gene starts [50,51,52,53,54]. Various functions have been suggested for non-canonical transcription factor binding sites, including acting as “decoys” to buffer the transcriptional response at regulatory targets [51,55], and mediating chromosome structure by forming long-range interactions with other DNA-bound transcription factors [56]. These are also possible functions of intragenic σ^54^ binding sites. We also propose that some intragenic σ^54^ binding sites are non-functional and thus represent biological noise. This is more likely for σ^54^ family members than σ^70^ family members since RNAP:σ^54^ requires a nearby bEBP in order to transcribe RNA. Genome-scale mapping of bEBP binding will be a powerful approach for distinguishing functional σ^54^ binding sites from non-functional sites since the likelihood of non-functional σ^54^ and bEBP binding sites being in close proximity is very low.

## Materials and Methods

### Strains and plasmids

All strains and plasmids used in this work are listed in S6 Table. All oligonucleotides used in this work are listed in S7 Table. *E*. *coli* MG1655 has been described previously [57]. Our laboratory version of MG1655 lacks an insertion element upstream of *flhD*, rendering the strain non-motile [18]. To generate *E*. *coli* MG1655Δ*rpoN* (RPB146), the Δ*rpoN*::*kan* ^*R*^ allele and flanking sequence was amplified by colony PCR using primers JW4588 and JW4589 from BW25113 Δ*rpoN*::*kan* ^*R*^ [58]. The PCR product was recombined into the chromosome of MG1655 Δ*thyA* containing plasmid pKD46 [59] by recombineering [60]. Following stable chromosomal integration and curing of pKD46, the *kan* ^*R*^ gene was resolved using with FLP-recombinase encoded on pCP20 and cured of the plasmid, as described previously [60]. Wild-type *thyA* was replaced at its native locus by P1 transduction from MG1655 (*thyA* ^+^) with selection on M9 minimal medium lacking thymine.

Derivatives of MG1655 with C-terminally 3x FLAG-tagged NagE (RPB220) and YqeB (RPB232) were generated using FRUIT [59]. Promoter mutants within the *nagB* (RPB277) and *yqeC* (RPB279) genes were constructed in the context of RBP220 and RPB232, respectively, using FRUIT [59].

*Salmonella enterica* subspecies *enterica* serovar Tyhpimurium strain 14028s [61] was used for *S*. *enterica* ChIP-qPCR.

Plasmid pBAD24 has been described previously [62]. Plasmid pRpoN was created by colony PCR amplification of the *E*. *coli rpoN* gene with primers JW3439 and JW3440 and cloning of this product using the InFusion method (Clontech) into the *Nco*I restriction site of pBAD24.

### Media and growth conditions

Cultures for ChIP-seq and RNA-seq were grown in M9 minimal media supplemented with 0.4% glycerol at 30°C with shaking (225 rpm) to mid-exponential phase (OD600 ~0.5). When necessary, the media was supplemented with 100 μg/mL ampicillin to select for plasmid retention. Arabinose was added to a final concentration of 0.2% for 10 minutes to strains carrying pRpoN or pBAD24 to induce over-expression of the *rpoN* gene, or as a negative control, respectively, for RNA-seq analysis. For analysis of intragenic promoter mutants under nitrogen limiting conditions (Fig 7), cultures were grown in Gutnick medium [63] at 30°C and supplemented with 2 mM NH_4_Cl. Cultures were harvested 60 minutes after growth ceased (nitrogen depleted), typically at an OD_600_ between 0.6 and 0.7. *S*. *enterica* cultures were grown in LB medium at 30°C to mid-log phase.

### ChIP-seq

ChIP-seq libraries were constructed as previously described [13] using MG1655. σ^54^ and RNAP were immunoprecipitated using 5 μL anti-σ^54^ or 1 μL anti-β antibody (Neoclone), respectively. Libraries were sequenced using a HiSeq 2000 sequencer (Illumina; University at Buffalo Next Generation Sequencing Core Facility). Alignment of sequence reads and identification of enriched regions (“peaks”) in the ChIP-seq data were performed as previously described [18]. “Fold Above Threshold” (FAT) scores indicate relative enrichment of regions in the ChIP-seq data, with a value of 1 being the threshold used to call peaks. To identify low stringency peaks, we reduced the threshold values 5-fold.

### RNA-seq

DNA-free RNA was prepared from two independent biological replicates of *E*. *coli* MG1655 Δ*rpoN* containing pBAD24 (RPB152) or pRpoN (RPB149), using the hot phenol method described previously [64]. rRNA was removed using the RiboZero kit (Epicentre) followed by preparation of strand-specific DNA libraries for Illumina sequencing using the ScriptSeq 2.0 kit (Epicentre). Libraries were sequenced as described above for ChIP-seq. Sequences were aligned to the *E*. *coli* MG1655 genome, and differences in expression between strains were determined using Rockhopper [65] with default settings.

### σ^54^ binding site conservation analysis

To find *E*. *coli rpoN* corresponding motifs in other species, we first created a Position Specific Scoring Matrix (PSSM) based on the alignment of all motifs found in *E*. *coli*. The PSSM was calculated by importing the nucleotide frequencies from each position in the motif using PSSM-convert [66]. This matrix was used to score the relative level of conservation of putative motifs found in other genomes of interest. To assess if each particular motif present in *E*. *coli* is also present and conserved in other corresponding genomes, we first screened for motifs that were found inside coding genes in *E*. *coli*. For this, we extracted a 300 nt fragment from the *E*. *coli* genome, centered on the position of the motif. We used BLASTX [67] to find if the query genomes contain a homologous protein using an E-value cutoff of 1e^-04^ (turning off the low complexity filter). From the genome position of the top BLAST hit, we calculated the PSSM score at the exact location where the motif is found in *E*. *coli* as well as any other potential conserved motif at alternate positions 100 bp upstream to 100 bp downstream from the *E*. *coli* position. For motifs that are located in non-coding regions, we first used BLASTN with a 300 bp fragment from *E*. *coli* to screen for the presence of homologous regions in the query genomes. If no hits were discovered, we refined the search by taking the corresponding gene-encoding sequence downstream of the motif in *E*. *coli*, and used BLASTX to search for a homologous protein. We used the position of the top hit to locate the corresponding position of the motif upstream of the gene. We then calculated the PSSM score for the exact location and at alternate positions using the same strategy from the in-ORF motifs. Conservation scores for the motifs were displayed using TreeView [68].

### σ^54^ binding site motif detection and positional analysis relative to ChIP-seq peaks

Motif enrichment analysis for ChIP-seq and ChIP-chip data was performed using MEME (default parameters for MEME-ChIP) [22,69]. For ChIP-seq data, 150 bp regions centered on the peak were used for MEME analysis. Centrimo (default parameters) was used to determine central enrichment of motifs [70]. Motif enrichment analysis for data from [28] was performed using MEME (default parameters for MEME-ChIP except that sequence was only searched on one strand) using regions 500 bp upstream of each gene.

### Phylogenetic conservation analysis for specific positions within intragenic σ^54^ binding sites

For each *E*. *coli* intragenic σ^54^ binding site, we counted the number of differences at “important” positions (positions 3, 4, 14, 15 and 16 from Fig 1B) and unimportant positions (positions 1, 8, 9, 10 and 12 from Fig 1B) between the *E*. *coli* site sequence and the sequence of equivalent sites in other species where the non-*E*. *coli* site had a PSSM score >6 and was perfectly aligned in the BLAST analysis. We did not examine important positions where the *E*. *coli* site differed from the consensus (example shown in S6B Fig).

### Analysis of RNAP occupancy at σ^54^ binding sites

RNAP (β) ChIP-seq sequence read counts were determined for the positions from -500 to +500 bp relative to each of the 135 high stringency σ^54^ ChIP-seq peaks (orientation defined by the associated σ^54^ binding site motif). All values were normalized to the value at position 0. “Median Occupancy Score” was calculated by determining the median value at each of the 1001 positions for all 135 high stringency σ^54^ ChIP-seq peaks. As a control we repeated this analysis using 135 randomly selected genome coordinates.

### qRT-PCR

RNA was prepared from nitrogen-depleted cultures as described for RNA-seq. RNA was reverse transcribed using SuperScript III reverse transcriptase (Invitrogen) with 150 ng random hexamer, according to the manufacturer's instructions. A control reaction, omitting reverse transcriptase, was performed. 0.5% of the cDNA (or negative control) was used as a template in a quantitative real time PCR using an ABI 7500 Fast real time PCR machine, with appropriate primers (S7 Table). Expression levels in the mutant strains were determined relative to wild type and normalized to expression of a control gene (*glnA*).

### Western blotting

Cell pellets from nitrogen-depleted cultures (1 mL) were resuspended in loading buffer based on OD600. Equal volumes were separated on a 4–20% acrylamide gradient gel (Bio-Rad). Proteins were transferred to PVDF membrane and probed with M2 mouse anti-FLAG antibody (Sigma; 1 in 2,000 dilution) or mouse anti-β’ antibody (Neoclone; 1 in 7,000 dilution), and HRP-conjugated goat anti-mouse antibody (1 in 10,000 dilution). Tagged proteins were visualized using the Clarity Western Substrate kit (Bio-Rad).

### ChIP-qPCR

ChIP and input samples were prepared as previously described [64]. For validation of putative σ^54^ binding sites in *E*. *coli* (MG1655 and RPB146) and *S*. *enterica* (14028s), cells were grown in LB medium. For NtrC-activating conditions, cells were grown in Gutnick medium with 2 mM NH_4_Cl. 2 μL anti-σ^54^ antibody (Neoclone) was used for ChIP in all cases. A “no antibody” control was performed in parallel for *S*. *enterica* since an isogenic deletion of the *rpoN* gene was not available. Enrichment was measured by quantitative PCR (qPCR) using an ABI 7500 Fast real time PCR machine, with appropriate primers (S7 Table). Enrichment was calculated relative to a control region within the *bglB* gene for MG1655, or STM14_2479 for *S*. *enterica*, and normalized to values for input DNA. Occupancy units were calculated as background-subtracted, fold-enrichment. Relative σ^54^ occupancy at *nagB* and *yqeC* intragenic promoters (Fig 7B) was calculated as the ratio of occupancy units to the value for the *glnA* promoter.

## Supporting Information

S1 Fig ChIP-seq peaks not associates with motifs are false positives. Targeted validation of five putative σ^54^ binding sites identified by ChIP-seq for which no associated motif was detected. ChIP-qPCR measurement of σ^54^ binding at putative sites identified by ChIP-seq in wild-type (MG1655; black bars) and Δ*rpoN* (RPB146; white bars) *E*. *coli* strains. Occupancy units represent background-subtracted enrichment of target regions relative to a control region within the transcriptionally silent gene *bglB*. Error bars represent the standard deviation from three independent biological replicates for wild-type cells, and two independent biological replicates for Δ*rpoN* cells.
(TIF)

S2 Fig ChIP-seq data for the σ^54^ binding site upstream of *crl*. σ^54^ and RNAP (β) binding as determined using ChIP-seq. The schematic depicts the local genomic environment surrounding *crl*. Grey arrows represent genes. The bent, black arrow indicates the location and direction of the σ^54^ binding motifs associated with identified ChIP-seq peak. Histograms show mapped sequence reads from σ^54^ (blue) and β (black) ChIP-seq experiments. Percentages indicate relative scale on the y-axis.
(TIF)

S3 Fig Characteristics of low stringency σ^54^ binding sites. **(A)** Consensus motif of 149 σ^54^ ChIP-seq peaks determined using MEME. **(B)** Centrimo analysis of σ^54^ motifs identified by MEME, showing the position of the motifs relative to the ChIP-seq peak centers. The graph indicates the average density of motif position for all 149 motif-containing regions, using 10 bp bins from position -75 to +75 relative to the σ^54^ ChIP-seq peak.
(TIF)

S4 Fig Comparison of σ^54^ binding sites between this study and [29]. **(A)** Highest-scoring consensus motifs derived from 94 σ^54^ ChIP-seq peaks unique to [29], determined using MEME (6, 6 and 7 regions contributed each of the three motifs, respectively). E-values determined by MEME are indicated. **(B)** Consensus motif derived from 75 σ^54^ ChIP-seq peaks unique to our study, determined using MEME (70 regions contributed to the motif). E-value determined by MEME is indicated. **(C)** Centrimo analysis of 70 σ^54^ motifs unique to our study, identified by MEME, showing the position of the motifs relative to the ChIP-seq peak centers. The graph indicates the average density of motif position for all 70 motif-containing regions, using 10 bp bins from position -75 to +75 relative to the σ^54^ ChIP-seq peak.
(TIF)

S5 Fig Comparison of σ^54^ binding sites between this study and [28]. Highest-scoring consensus motifs derived from 22 using MEME (14, 10 and 2 regions contributed each of the three motifs, respectively). E-values determined by MEME are indicated.
(TIF)

S6 Fig Analysis of sequence conservation at “important” and “unimportant” positions within intragenic σ^54^ binding sites. **(A)** Sequence alignment of the IS46 σ^54^ binding site from *E*. *coli* (inside the *wecA* gene) with sequences the equivalent position in homologous genes in other bacterial species. Differences to the *E*. *coli* sequence are highlighted in gray. The PSSM score, a measure of how well a sequence matches the expected motif, is shown for each site. The number of differences between *E*. *coli* and non-*E*. *coli* species are shown for each of the five “important” base positions (i.e. those that have a strong consensus base; red text), and each of the five “unimportant” base positions (i.e. those with low information content in the PSSM; blue text). **(B)** As above, but for site IA27 (inside the *istR* gene). Note that position 16 of this site does not match the consensus and therefore was excluded from the analysis. **(C)** Summary of the number of substitutions seen for each of the important and unimportant base positons for all comparisons. For each pair of numbers, the first number indicates the number of substitutions observed, and the second number indicates the number of comparisons examined.
(TIF)

S1 Table List of ChIP-seq peaks from the high stringency analysis, not associated with a motif identified by MEME. (XLSX)

S2 Table List of ChIP-seq peaks identified only using the reduced threshold. (XLSX)

S3 Table Comparison of high stringency ChIP-seq peaks with putative σ^54^-bound regions identified by [29]. (XLSX)

S4 Table Analyzed RNA-seq data for all genes that are significantly regulated (*q* < 0.01, fold change > 2) following transient overexpression of σ^54^. (XLSX)

S5 Table Conservation of all high stringency σ^54^ binding sites across a range of bacterial species. (XLSX)

S6 Table List of strains and plasmids used in this study. (XLSX)

S7 Table List of oligonucleotides used in this study. (XLSX)
